# High- and Low-Fluorescent Photoinitiators for Multiphoton Lithography

**DOI:** 10.1021/acsapm.5c01802

**Published:** 2025-07-31

**Authors:** Dimitra Ladika, Michalis Stavrou, Gordon Zyla, Kostas Parkatzidis, Maria Androulidaki, Frederic Dumur, Maria Farsari, David Gray

**Affiliations:** † Institute of Electronic Structure and Laser, Foundation for Research and Technology-Hellas, 70013 Heraklion, Greece; ‡ Laser Research Center, Physics Faculty, 54694 Vilnius University, Vilnius LT-10223, Lithuania; ¶ Department of Chemical Engineering, 6429 Stanford University, Stanford, California 94305, United States; § Aix Marseille University, CNRS, ICR, UMR 7273, F-13397 Marseille, France

**Keywords:** Multiphoton lithography, polymerization, photoinitiators, fluorescence, nonlinear optical properties

## Abstract

Multiphoton lithography (MPL), an additive manufacturing method, enables the fabrication of intricate three-dimensional micro- and nanostructures with high spatial resolution, crucial for applications in photonics, micro-optics, and biomedicine. Central to the performance of the MPL is the choice of photoinitiator (PI), which governs polymerization efficiency, resolution, and application-specific functionality. However, conventional PIs often suffer from drawbacks such as high autofluorescence and poor spectral selectivity, limiting their utility in fluorescence-sensitive applications. This work presents a systematic study on the nonlinear optical (NLO) properties of lab-made low-fluorescence PIs (LF, indane-1,3-dione-based push–pull compounds), comparing them to high-fluorescence PIs (HF, triphenylamine-based aldehydes), and examines their effectiveness for MPL. The NLO properties of the PIs were investigated employing the two-beam initiation threshold (2-BIT) method and Z-scan technique both in solution and integrated into the hybrid photoresist SZ2080. The characterization of NLO properties and manufacturing tests were performed within a single optical setup, under similar spectrotemporal laser radiation conditions (pulse width, 150 fs; wavelength, 780 nm). This proposed approach allows for a straightforward and efficient evaluation of the suitability of aPI for MPL. LF-PIs were found to be up to 2 orders of magnitude less fluorescent than HF-PIs, as determined by photoluminescence analysis, and exhibited up to 10-fold higher NLO absorption-related parameters. This indicates that high fluorescence may compete with the NLO performance by interfering with absorption processes essential for effective polymerization. Most importantly, LF-PIs enabled structuring performance comparable to that of SBB (a benchmark material for low-fluorescent MPL-fabricated structures) when embedded in SZ2080, and the resulting printed structures exhibited an improved selective fluorescence response, indicating their strong potential for printing scaffolds in biorelated applications, where a high fluorescent signal usually hinders signal detection and analysis.

## Graphical abstract

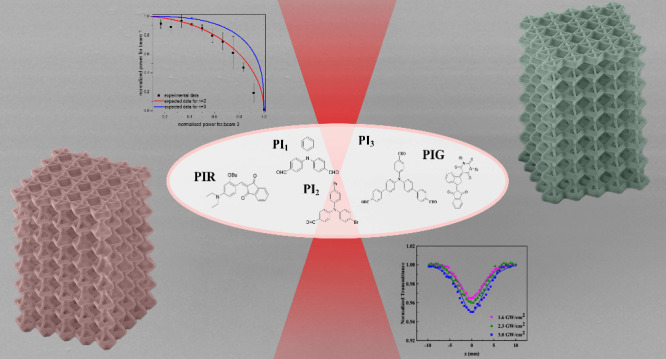

## Introduction

The interaction between light and matter plays a key role in many physical and chemical processes, specifically enabling advancements in laser-based 3D printing techniques.^1^ Among these, multiphoton lithography (MPL) provides maskless fabrication of true 3D micro- and nanostructures with intricacy and high precision.^2,3^ Due to these capabilities, MPL has emerged over the past two decades as a promising and versatile 3D printing technique for applications requiring high-resolution features and intricate geometries, such as photonics,^1^ optoelectronics,^4^ micro-optics,^5^ bioimaging,^6^ and tissue engineering,^7^ among others.^8^

To further advance MPL, current trends focus on increasing the efficiency of both the process and the fabricated structures through improvements in optical setups and the development of improved photosensitive materials.^9–13^ For the latter, a crucial factor to consider is the selection of the photoinitiator (PI) and its photophysical properties, as the PI serves as a seed, primarily triggering the polymerization process by generating free radicals that initiate the cross-linking of monomers.^8,14,15^ In particular, even a small amount of the PI in the host photoresist can influence the absorption depth *l*_*s*_ (*l*_*s*_ = 1/α, where α = α_0_ + β*I*, with α_0_ as the linear absorption coefficient, β as the two-photon absorption coefficient, and *I* as the light intensity), leading to enhanced light energy deposition. This enhancement arises from an increase in α, driven by the intensity-dependent nature of multiphoton absorption (MPA), ultimately facilitating photopolymerization.^16^ From this perspective, key characteristics in MPL (such as feature resolution, primarily determined by voxel size and scanning speed, and the overall quality of the 3D-printed structure) critically depend on the choice of PI. However, beyond absorption characteristics, several other factors critically affect the initiation efficiency in multiphoton polymerization. These include the excited-state dynamics of the PI (intersystem crossing and nonradiative decay),^17^ radical reactivity, fragment mobility within the prepolymer matrix, spatial distribution of deposited energy, and propagation kinetics, all of which synergistically govern the radical formation and propagation.^18^

In general, most of the PIs, often based on polycyclic aromatic hydrocarbons, tend to be toxic and exhibit strong autofluorescence and intrinsic coloration. These properties can compromise environmental sustainability and obscure signal detection in sensitive applications, such as confocal and two-photon microscopy^19^ or tissue engineering.^20^ The autofluorescence properties of PIs can influence their efficiency in MPL. High autofluorescence may reduce efficiency if a substantial portion of the excitation energy is dissipated through radiative decay rather than contributing to polymerization initiation, due to competing relaxation pathways. However, this is not always the case, as other nonradiative deactivation pathways can also affect overall efficiency.^21^ Nevertheless, several high fluorescent PIs can be beneficial in imaging applications. In particular, studies have demonstrated that these high fluorescence properties not only enhance energy transfer for photoinitiation but also provide efficient long-wavelength light absorption.^22,23^ Therefore, it is inferred that depending on the application, 3D structures fabricated with either high or low fluorescence can be used to achieve specific functionalities.

For example, rhodamine 6G is a high fluorescent PI that has been employed in applications such as *in situ* monitoring of polymerization or microfluidics mapping.^24,25^ In contrast, Sudan black B (SBB), a commercially available dye, proved to be efficient as low-fluorescent PI as it suppresses, at large degree, unwanted fluorescence in visible wavelength.^26,27^ However, the low broadband fluorescence of SBB may be a drawback for applications requiring selective wavelength emissions, such as multicolor single-molecule localization microscopy, where even weak broadband background signals can hinder precise spectral separation and molecular localization.^28^

To address the issues associated with PIs (either low or high fluorescent), an overall solution may be the use of nonphotosensitized resists, as ultrafast lasers deposit considerable energy, enabling polymerization without the need for a PI, which has been reported in several works.^29–32^ However, while this approach offers certain benefits for some materials, it remains inefficient for widespread application in material processing, as the processing efficiency depends on the material’s natural absorption properties and the laser wavelength used. Additionally, in the absence of a PI, radicals are generated directly from the material or from oxygen molecules. This leads to an uncontrolled process, causing higher diffusion and ultimately resulting in a lower resolution. Thus, in respect to the most commonly used ultrafast lasers for MPL, which typically emit around 800 nm, the use of PIs remains beneficial, as they can enhance MPA, improve spatial resolution by providing wider fabrication windows, and ultimately ensure the highest MPL efficiency.^3,16^

Indeed, the highest efficiency for MPL can be achieved when the PI triggers two-photon absorption (TPA), as this requires the lowest laser peak intensity to initiate nonlinearly induced photopolymerization.^33^ Linear characterization approaches, such as UV–vis spectroscopy, which allow the study of a PI’s absorption properties, can initially help identify a suitable candidate. However, to gain deeper insights into the effectiveness of a PI and the processes that take place in a PI upon intense laser radiation, nonlinear approaches are required. For instance, indirect methods such as exposure-time methods, the line-width method, or the two-beam initiation threshold method (2-BIT) allow the determination of the effective order of nonlinear absorption (*n*),^34^ while direct methods such as two-photon excited fluorescence or Z-scan can determine the nonlinear absorption coefficient β and TPA cross-section σ.^35^

A comprehensive understanding of MPL using different PIs requires careful consideration of the methods used to assess their effectiveness. Both indirect and direct methods present limitations that can hinder the accurate evaluation of their ability to initiate oligomer cross-linking. Indirect methods, which investigate PIs already incorporated into the photoresist, more closely resemble actual MPL scenarios. However, because these methods rely on observable events like changes in sample’s transmittance, they can be sensitive to human perception factors, such as visual capabilities. In contrast, direct methods usually isolate the PI from the complex composition of the resist, focusing instead on PI properties such as the two-photon absorption cross-section.^36^ While these methods provide valuable data, they may not fully capture the behavior of the PI within the photoresist. Functionalization of the photoresist upon mixing with the PI^32^ can modify its nonlinear absorption and refraction, which are not accounted for in direct studies of PI alone. Thus, reliance on either direct or indirect methods alone leads to an incomplete picture, highlighting the importance of a combined approach.

Given the advantages and limitations of each approach, combining both methods proves to be the most effective strategy for studying PIs nonlinearly. Interestingly, this can be achieved within a single optical setup typically used for MPL, allowing for a straightforward evaluation of the PI efficiency. Leveraging this integrated setup, the present study primarily aims to evaluate the suitability of five photoinitiators for MPL, two low-fluorescence (LF) indane-1,3-dione-based push–pull compounds, and three high-fluorescence (HF) triphenylamine-based aldehydes with varying aromaticity,^37^ as PIs for MPL either dissolved in solvents or integrated into the commercially available hybrid organic–inorganic SZ2080.^38,39^ Their linear and nonlinear properties are analyzed using UV–vis spectroscopy, the 2-BIT^40^ method, and the Z-scan technique and compared with standard LF-PIs and HF-PIs in MPL, namely, Michler’s ketone (BIS)^41^ and Sudan black B (SBB),^42^ respectively. By integrating these nonlinear optical characterization methods within a single experimental setup, a comprehensive approach is presented for assessing PI suitability prior to MPL. This enabled the identification of promising alternatives to conventional LF-PIs (i.e., SBB), thereby addressing the limited availability of suitable LF-PIs for MPL. Notably, one of the LF-PIs (later referred to as PIG) is introduced here for the first time in the context of 3D printing using MPL. It exhibits nonlinear optical properties comparable to those of SBB while enabling the fabrication of high-quality and well-defined 3D-printed structures.

## Materials and Methods

### Photoinitiators

All reagents and solvents were purchased from Sigma-Aldrich, Germany, or Alfa Aesar, United States, and used as received without further purification. Elemental analyses were recorded with a Thermo Finnigan EA 1112 elemental analysis apparatus driven by the Eager 300 software. ^1^H and ^13^C NMR spectra were determined at room temperature in 5 mm o.d. tubes on a Bruker Avance 400 spectrometer of the Spectropole: ^1^H (400 MHz) and ^13^C (100 MHz). All ^1^H chemical shifts were referenced to the solvent peak DMSO-*d*_6_ (2.49 ppm), and the ^13^C chemical shifts were referenced to the solvent peak DMSO-*d*_6_ (39.5 ppm). The chemical structures of the PIs are schematically presented in Figure 4A, and their syntheses are described below (with further details provided in the Supporting Information):

- HF-PIs: Triphenylamine-based aldehydes with varying aromaticity (denoted as PI_1_, PI_2_, and PI_3_) arising through formyl group, Br, and benzaldehyde electrophilic substitution have been previously reported in literature, without modifications and similar yields.^41,43,44^

- LF-PIs: The structures of the synthesized LF-PIs were named as indane-1,3-dione-based compounds, functionalized with 2-thioxodihydropyrimidine and 2-butoxy-4-dimethyl­aminobenzene electron donating groups (denoted as PIG and PIR). While PIR has been previously reported by Pigot et al.,^45^ PIG is introduced here for the first time. The synthesis procedure is as follows: Indane-1,3-dione (2 g, 13,68 mmol, 146.15 g/mol) and 1,3-diethyl-2-thiobarbituric acid (2.74 g, 13,68 mmol, *M* = 200.26 g/mol) were suspended in absolute ethanol (50 mL), and a few drops of piperidine were added. The mixture was placed in a preheated oil bath at 100 °C and refluxed overnight. During reflux, a yellow precipitate (CH_26_H_20_N_2_O_4_S) formed. After cooling to room temperature, the solution was acidified with diluted aqueous HCl. The resulting yellow solid was filtered, washed several times with ethanol and then with pentane, and dried under vacuum (43% yield). NMR analyses were performed in DMSO-*d*_6_, in which only the anionic form of PIG was detected, as is typically observed for strong electron acceptors in this solvent.

The selection of indane-1,3-dione and aldehyde cores as the building blocks for synthesizing effective PIs was driven by their multiple aspects for chemical functionalization and electrophilic substitution such as oxygen atoms and aromatic rings. These building blocks were leveraged to enhance π-electron delocalization and promote the formation of efficient donor−π–acceptor (D-π-A) charge transfer systems.

### Materials Synthesis

For MPL and 2-BIT, the PIs were mixed within non-photosensitized variants of the Zr-based hybrid organic–inorganic photoresist called SZ2080^TM^, which is now widely used in the field of MPL. Specifically, the photoresist is composed of methacryloxypropyl trimethoxysilane (MAPTMS, 97%) and 2-(dimethylamino)­ethyl methacrylate (DMAEMA, 98%), which build the organic photopolymerizable monomers, while zirconium *n*-propoxide (ZPO, 70%) and the alkoxysilane groups of MAPTMS form the inorganic network. The resist was synthesized via the sol–gel process by using the following molar ratios of the components: MAPTMS:ZPO = 8:2 and (MAPTMS + ZPO):DMAEMA = 3:1. All of the chemical components, as well as the commercially available PIs, BIS and SBB, were purchased from Sigma-Aldrich, Germany. Prior to the addition of the PIs into the photoresist, they were dissolved in dichloromethane (DCM) at a 1% w/v concentration. Afterward, all solutions, except the one containing the SBB as PI, were incorporated onto the photoresist in a 1% w/w concentration, with respect to the monomers. SBB solution was added in the photoresist in a 0.3% w/w concentration, with respect to the monomers. The final mixture was filtered using a 0.22 μm pore size syringe filter and left for 4 h in vacuum for solvent evaporation.

### Method for the Linear Characterization of HF- PIs and LF-PIs

#### UV–Visible–NIR Absorption Spectra

To measure the linear absorption of the PIs, 1 mg of powder of each PI was dissolved in 1 mL of DCM, resulting in different molecular concentrations; PI_1_ (6.6 mM), PI_2_ (4.6 mM), PI_3_ (6.1 mM), PIR (2.6 mM), PIG (2.5 mM), BIS (6.2 mM), and SBB (2.2 mM). Then, the PerkinElmer model Lambda 25 UV–vis–NIR double beam spectrophotometer was employed to record the absorption spectra of the prepared solutions. All spectroscopic measurements were recorded across a wavelength range of 300 to 1000 nm, with the solutions placed in 1 mm thick glass cells. Notably, the spectrum of DCM was subtracted from the spectra of the measured solutions to remove background absorption.

#### Photoluminescence

Photoluminescence analysis of MPL-printed cubes (dimensions: 120 × 120 × 15 μm^3^) from the PIs incorporated into SZ2080^TM^ was conducted using a He–Cd CW-laser at 325 nm with a laser power of 35 mW. The laser beam was focused using a fused silica lens with a 4 mm focal length, resulting in a minimum spot size of 80–100 μm.

### Methods for Nonlinear Characterization of HF- PIs and LF-PIs

#### 2-BIT Method

The 2-BIT approach was designed as shown in Figure 1, using the same system for both nonlinear characterization and MPL. A femtosecond fiber laser system (FemtoFiber ultra 780, Toptica Photonics AG) was used, emitting at 780 nm with a pulse duration of 150 fs and a repetition rate of 80 MHz. The emitted laser beam was divided into two equal parts through a 50:50 nonpolarizing beam splitter, as 2-BIT requires two temporally and spatially overlapped laser beams to determine the effective order of multiphoton absorption (*n*) of the PIs when incorporated into the photoresist. Notably, the two laser beams were identical in wavelength, pulse duration (τ), and repetition rate (*f*), but differed in intensity. The timing of the beams was adjusted so that pulses arrive at the sample at a repetition rate of 2*f* (160 MHz), where *f* is the fundamental repetition rate of the laser (80 MHz). Finally, the overlapped beams were focused into the sample using a high numerical aperture focusing microscope oil-immersion objective lens (100×, NA = 1.4, Zeiss, Plan Apochromat), with its back aperture overfilled. The sample was mounted on an *XYZ* piezoelectric stage system (Physik Instrumente M-110.1DG, Germany).

**Figure 1:**
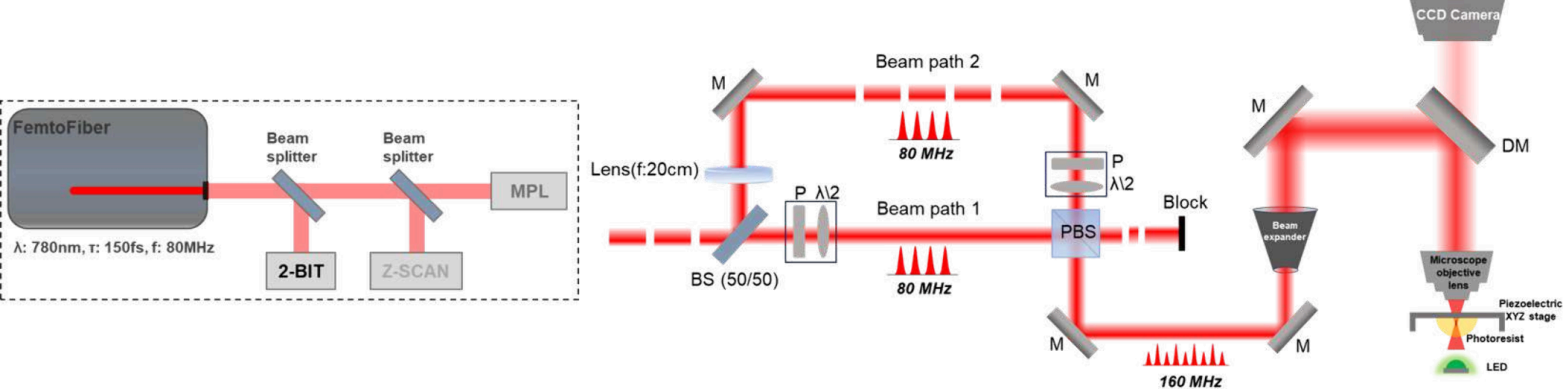
2-BIT/Z-scan/MPL optical system (left) and a detailed schematic of the 2-BIT experimental setup (right): BS, beam splitter; P, polarizer; λ/2, half-wave plate; PBS, polarizer beam splitter; M, mirror; DM, dichroic mirror.

As part of the 2-BIT measurements, the minimum power at which polymerized features became visible for each beam (*P*_th_) was determined by using the same procedure. In this process, one axis of the piezoelectric stage moved horizontally at a constant velocity of 20 μm/s, while the laser power for one beam path was incrementally increased until polymerization became observable in real-time using a CCD camera, with the other beam blocked.

Subsequently, to assess the value of *n* for the mentioned photoresist incorporating different PIs, the power of the first beam was varied in regions below the polymerization threshold. In contrast, the power of the second beam was adjusted until polymerization was observed, following the same procedure as previously described, using one axis of the moving piezoelectric stage. Once polymerization was visually detected, the power values of both beams were recorded.

To enable a quantitative analysis of *n*, the power of the first beam was adjusted in at least ten increments. Additionally, three sets of fresh samples were measured for each PI.

Importantly, the threshold power serves as a normalization reference throughout the entire 2-BIT procedure, ensuring reproducibility regardless of the optical system.

To ultimately determine the value of *n*, the mathematical expression describing the exposure threshold required to initiate polymerization in a photosensitive material via MPL is given by the sum of the powers of each laser beam, as defined in eq 1:

1 $$P_{1}^{n}+P_{2}^{n}=P_{\mathrm{th}}^{n}$$

Equation 1 can also be rewritten in terms of normalized powers, from which *n* can be directly calculated. With ${\mathrm{P̅}}_{1}=P_{1}/P_{\mathrm{th}}$ and ${\mathrm{P̅}}_{2}=P_{2}/P_{th},$ respectively, eq 2 becomes

2 $${\mathrm{P̅}}_{1}=\sqrt[{n}]{1-{\mathrm{P̅}}_{2}^{n}}$$

#### Z-Scan Technique

Z-scan is a well-established method for investigating the third-order optical nonlinearities of a sample by measuring its normalized transmittance under a focused laser beam, typically with a Gaussian intensity distribution, while the sample is translated along the beam’s propagation axis, for instance, using a high-precision stepper motor.^35,46^ More precisely, the studied material was exposed to different levels of laser radiation intensity, as it was driven along the propagation direction of a focused laser beam (which has a Gaussian intensity profile in this case) by a high precision stepper motor. The variation in the sample’s transmittance was monitored by two distinct experimental configurations known as “open-aperture” (OA) and “closed-aperture” (CA) Z-scans, which provide insights into its NLO absorption and refraction, respectively. Since the primary focus of this study was to evaluate the efficiency of various compounds for initiating polymerization via MPL, only the OA Z-scan configuration was employed to study their NLO absorption. To determine the nonlinear absorption coefficient β, the plot of the transmittance as a function of the sample position (*z*) was fitted using eq 3,^35^

3 $$T\left(z\right)=\frac{1}{\sqrt{\pi }\left(\frac{\beta I_{0}L_{eff}}{1+{\left(\frac{z}{z_{0}}\right)}^{2}}\right)}\int_{-\infty }^{+\infty }\ln\left[1+\frac{\beta I_{0}L_{eff}}{1+{\left(\frac{z}{z_{0}}\right)}^{2}}e^{-t}\right]dt$$

where $L_{\mathrm{eff}}=\frac{1-\exp\left(\alpha_{0}L\right)}{\alpha_{0}}$ represents the sample’s effective length, with α_0_ (cm^–1^) and *L* (cm) denotes the absorption coefficient at the excitation wavelength and the sample’s length, respectively. *I*_0_ (W cm^–2^) is the laser intensity at the focal plane, *z*_0_ (mm) is the Rayleigh length, and *z* (mm) indicates the sample’s position. Then, from the determined values of β, the imaginary part of the third-order susceptibility (Im χ^(3)^) is calculated through eq 4 below:

4 Im⁡χ(3)⁡(esu)=10−7c2n02β96π2ω

where *c* (m/s) is the speed of light, *n*_0_ is the refractive index, and ω (s^–1^) is the laser frequency. However, since Im χ^(3)^ represents a macroscopic quantity dependent on the solute concentration, the imaginary part of the second-order hyperpolarizability, Im γ, is frequently preferred. This parameter, being a molecular constant, describes the NLO absorptive response per molecule, thereby facilitating comparisons to other materials. The values of Im γ can be derived from Im χ^(3)^, using eq 5:

5 Im⁡γ⁡(esu)=Im⁡χ(3)NL4

where *N* is the number of molecules/cm^3^ and *L* = (*n*_0_^2^ + 2)/3 is the local field correction factor. In addition, the TPA cross-section σ can be determined from the obtained values of the nonlinear absorption coefficient β, employing eq 6 below:

6 $$\sigma =\frac{hv\beta }{N_{A}\rho }$$

where *h* [in J·s] is Plank’s constant, *N*_A_ [in mol^–1^] is the Avogadro number, and ρ [in mM] is the molecular density. The σ is measured in the Goeppert Mayer (GM) units (1 GM = 10^–50^ cm^4^·s·photon^–1^).

A schematic configuration of the OA Z-scan experimental apparatus is shown in Figure 2. The laser beam was focused into a 1 mm thick glass cell (*l*) containing solutions of the PIs (see section Materials Synthesis) by a 20 cm focal length quartz plano-convex lens. Afterward, the entire transmitted laser beam was collected by a second lens and recorded by a photodetector. The beam radius, *w*_0_, at the focal plane was measured by a CCD camera and found to be approximately (18 ± 0.2) μm (at 1/e^2^ maximum intensity). Thereby, the corresponding value of the Rayleigh length, given by $z_{0}=\frac{\pi {w_{0}}^{2}}{\lambda },$ is about 1.61 mm, which satisfies the thin sample approximation requirement (i.e., *l* < *z*_0_) of the Z-scan technique. To accurately determine the NLO absorption related parameters of the present photo initiators, Z-scan experiments were conducted for each compound at two different solution concentrations (i.e., 0.5 and 1 mg/mL).

**Figure 2:**
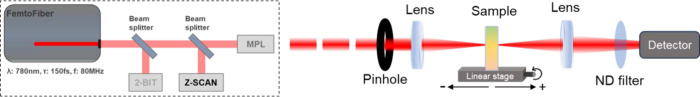
2-BIT/Z-scan/MPL optical system (left) and a detailed schematic of the Z-scan experimental setup (right). ND: neutral density filter.

### Multiphoton Lithography

#### Sample Preparation

Samples were prepared by drop-casting the photosensitive material onto silanized glass substrates (thickness: 0.14 mm) and kept under low-vacuum conditions overnight to allow gelation as the solvent evaporated. After MPL, the droplets were developed in 4-methyl-2-pentanone for 30 min to remove the unpolymerized resin.

Silanization involved the addition of MAPTMS monomers to the glass surface, enabling the printed micronanostructures to adhere to the substrate’s surface during photopolymerization. Typically, the process begins by immersing the glass substrate in ethanol and using ultrasound for 1 h to thoroughly clean the surface. Then, the substrate is placed in a solution containing 20 mL of DCM and 250 μL of MAPTMS and undergoes sonication for 4 h, promoting the attachment of MAPTMS chains to the surface.

#### Fabrication of 3D Microstructures

MPL was performed in a layer-by-layer manner using various structural designs, which were created with computer-aided design software (Fusion 360, Autodesk) and further processed by using slicing software (Istos, Biomimetics). In this process, hatching lines were spaced 0.2 μm apart, while layers were separated by 0.4 μm.Experimentally, the fabrication of the 3D microstructures was carried out using the setup shown in Figure 3, part of the same system used for NLO characterization. The fabrication parameters were controlled via commercial software Arachne (Biomimetic, Greece). The laser beam was tightly focused onto the sample through a microscope objective lens (20×/NA 0.8, Plan-Apo, Zeiss, Germany), while an LED provided illumination for real-time monitoring via a camera. An acousto-optical modulator (MTS40-A3-750.850, AA Opto Electronics, France) functioned as a shutter and a laser power modulator. A 2D galvanometric scanner (HurryScan II 10, Scanlab, Germany) enabled precise beam deflection within a single plane to produce single layers. After each layer was fabricated, the sample was moved along the vertical axis using a linear stage (M-605.1DD, Physik Instrumente, Germany). Additionally, two more linear stages of the same model allowed for the fabrication of multiple structures on a single sample.

**Figure 3:**
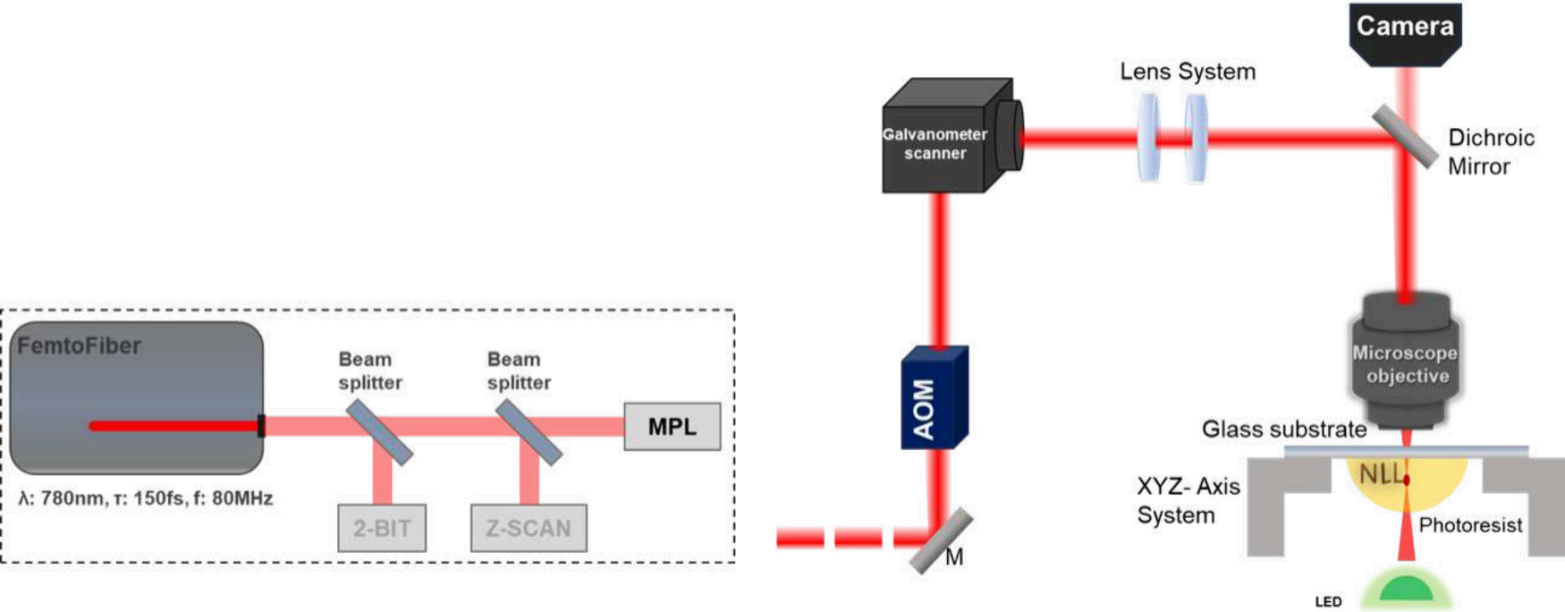
The 2-BIT/Z-scan/MPL optical system (left) and a detailed schematic of the MPL experimental setup (right): AOM, acousto-optic modulator; M, mirror.

## Results and Discussion

### Linear Characteristics of HF-PIs and LF-PIs

#### UV–Visible–NIR Absorption Spectra

In Figure 4, the UV–vis–NIR absorption spectra of HF-PIs (Figure 4B) and LF-PIs (Figure 4C) solutions are presented, normalized to their respective maximum absorbance. The colors of the plot lines for every PI represent the intrinsic coloration that they naturally exhibit, i.e., shades of yellow for PI_1_,PI_2_,PI_3_ and BIS, red for PIR, green for PIG, and black for SBB. As shown in Figure 4E, HF-PIs exhibit an absorption band centered between 360 and 380 nm, similar to Michler’s ketone, attributed to π–π* transitions of the aromatic rings, while remaining fully transparent throughout the visible and near-infrared regions. In contrast, LF-PIs display absorption bands in both the UV and visible spectral regions, most likely resulting from π–π* transitions, and intramolecular charge transfer (ICT): from 2-thioxodihydropyrimidine to indane-1,3-dione for PIG and from -butoxy-4-dimethylamino­benzene to indane-1,3-dione for PIR. The presence of efficient ICT in LF-PIs can be verified by their low fluorescence signal (see Figure 7B), as charge transfer can typically divert energy from fluorescence pathways. The spectra of LF-PIs display a red-shift compared with the corresponding spectra of HF-PIs, indicative of a larger π electron delocalization, which could effectively increase energy deposition in MPL.

**Figure 4:**
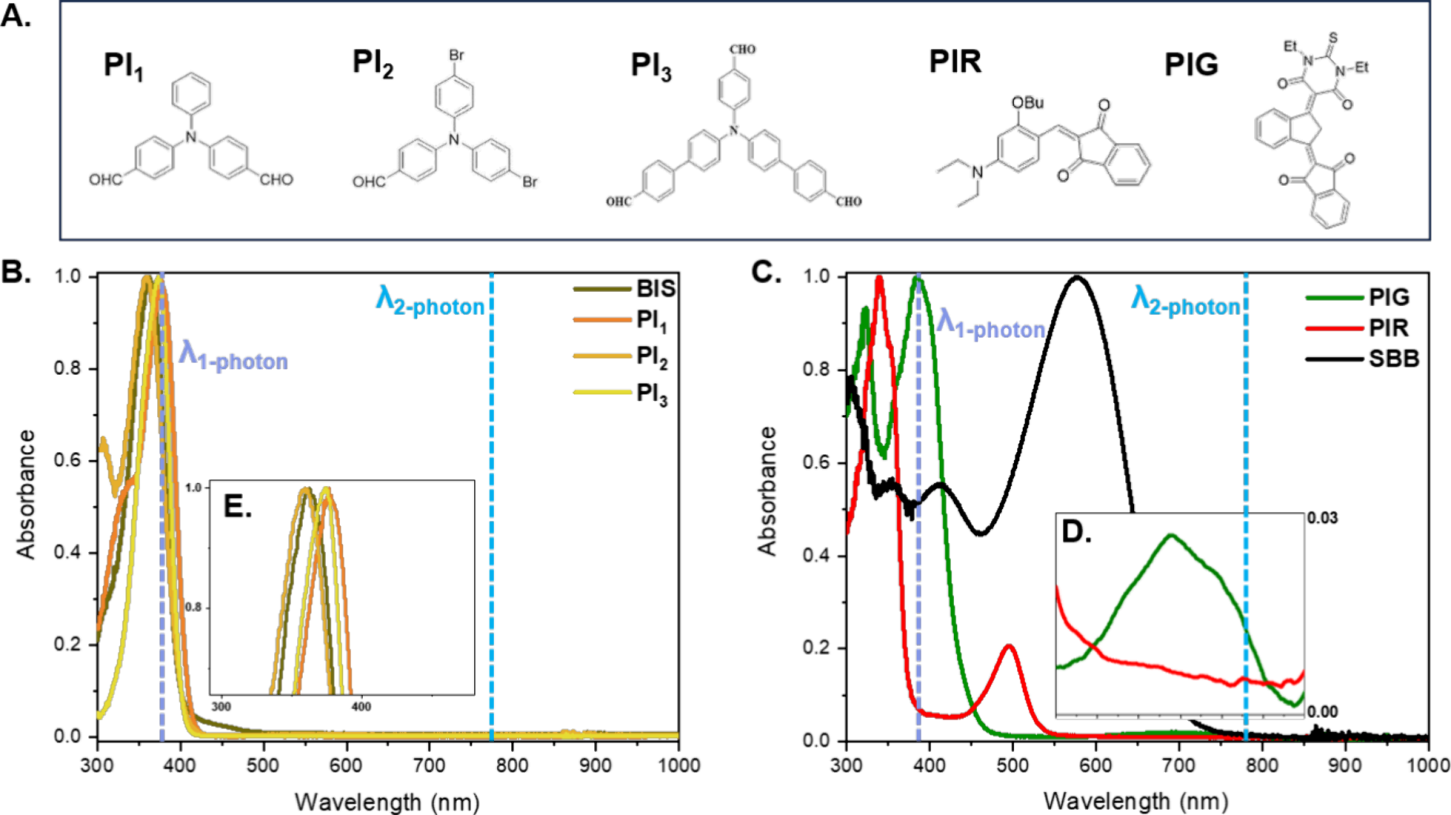
(A) Chemical structures of the investigated PIs. (B) Absorption spectra of HF-PIs and (C) LF-PIs in DCM. The dashed cyan and light-blue lines represent the wavelength for one- and two-photon absorption, correspondingly. (D) Close-up view of the absorption spectrum for PIR and PIG, highlighting the 550–850 nm range.

The linear absorption spectrum of PIR resembles those of the HF-PIs, with two peaks observed at 340 and 495 nm. Conversely, PIG displays a distinct linear absorption pattern compared to typical PIs used in MPL,^9,41^ with two peaks observed at 320 and 385 nm. As shown in Figure 4C, both LF-PIs and SBB exhibit absorption edges that extend into the visible spectral region, suggesting narrower energy bandgaps. This is characteristic of a greater π-electron delocalization and is associated with enhanced nonlinear absorption, which is beneficial for two-photon polymerization. However, it is worth noting that PIG displays a weak but noticeable linear absorption around 700 nm, indicating that its polymerization performance may not be exclusively attributed to two-photon absorption, as discussed in the rest of the article.

### Nonlinear Characterization of the PIs

#### 2-BIT Measurements

Prior to the discussion of the 2-BIT results, it is important to note that the absorption spectrum of the PIs may differ slightly in the photoresist. This effect can be attributed to functionalization of the host photoresist, as previously observed for BIS integrated into SZ2080^TM^ in the work by Stavrou et al.^32^ Spectra obtained in solvents offer only a semiquantitative approximation of the absorption band positions. In this context, experimental data from 2-BIT address this gap, as they were collected through *in situ* measurements of the photoresist sample, including each individual PI. Furthermore, a simple inspection of the absorption spectra morphology cannot rule out the manifestation of higher-order (*n* > 2) nonlinearities.

2-BIT measurements were conducted to determine the effective order of the nonlinear absorption (*n*) of the PIs. Figure 5 presents the 2-BIT results of HF-PIs and LF-PIs. For each experimental curve (solid points), the corresponding theoretical curve (solid lines) was numerically calculated using eq 2, considering contributions from one-, two-, and three-photon absorption. As can be seen, the experimental data for HF-PIs and PIR are in excellent agreement with the theoretical plots obtained for *n* = 2, denoting NLO absorptive response attributed to TPA. On the other hand, the corresponding data for PIG fall between the one- and two-photon regimes. For the accurate estimation of the experimental values of *n* for each PI, the raw data were fitted to eq 2, using the single-beam power threshold shown in Table 1. The determined values are gathered in the same table.

**Figure 5:**
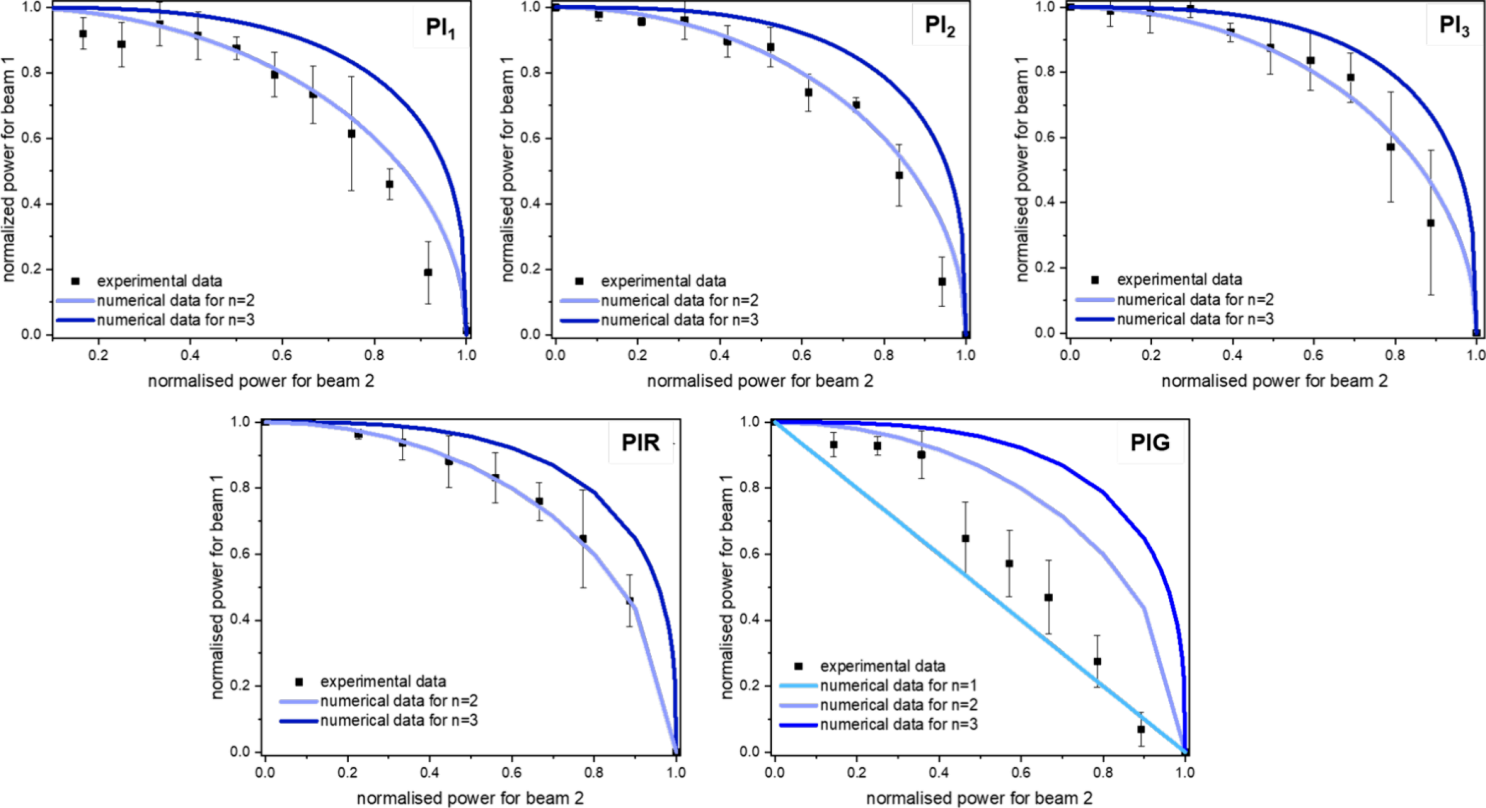
2-BIT data for the HF-PIs (PI_1_,PI_2_,PI_3_) and LF-PIs (PIR,PIG). The blue, light-blue, and cyan colored lines are the best fits to eq 1 for three-, two-, and one-photon absorption, respectively, given as a reference. The error bars represent standard deviations derived from multiple measurements.

**Table 1:** Effective Order of Nonlinear Absorption (*n*), the Single-Beam Power Threshold *P*_th_, and the Peak Intensity Threshold *I*_th_ of the HF- and LF-PIs

	PI_1_	PI_2_	PI_3_	PIG	PIR
*n*	1.9 ± 0.1	2.0 ± 0.1	2.0 ± 0.2	1.5 ± 0.2	2.1 ± 0.3
*P*_th_ (mW) ± 0.1	6.3	8.7	6.4	7.3	7.8
*I*_th_ (TW/cm^2^)	0.22	0.31	0.22	0.25	0.27

Building upon this, an analysis of the 2-BIT results for the HF-PIs is presented. Specifically, both PI_1_ and PI_3_ show strong absorption at approximately 385 nm (see Figure 4B), suggesting a quadratic response. This is confirmed by the best-fit exponents from the 2-BIT data, with values of *n* = 1.9 for PI_1_ and *n* = 2.0 for PI_3_, showcasing that both compounds exhibit quadratic behavior at 780 nm. Similarly, for PI_2_, the best-fit exponent *n* is equal to 2.0, indicating TPA. However, since the absorption band of PI_2_ has a peak at approximately 360 nm, the transitions through TPA are off-resonant, as the expected two-photon resonance wavelength (2 × 360 nm = 720 nm) differs from the excitation wavelength of 780 nm.

The 2-BIT results of the photoresist containing one of the LF-PIs, named PIR, showed a dominant effective nonlinear absorption of two-photon (i.e., *n* = 2.1). Therefore, given that the two absorption bands of PIR are centered at approximately 340 and 495 nm, and the determined effective order indicates the occurrence of TPA, this implies that TPA under 780 nm laser irradiation takes place in an off-resonant regime. Thus, PI_1_, PI_2_, PI_3_, and PIR produce radicals via two-photon absorption in both the resonant and off-resonant regime.

Although linear absorption spectra can offer some indication, predicting the effective order of nonlinear absorption remains challenging due to varying nonlinearities and the potential influence of selection rules on multiphoton transitions.^47^ These factors can be affected, for instance, by the molecular system, as seen in the cases of PIR and PIG, which exhibit multiple absorption bands. Nevertheless, the consistent trends observed in these PIs support the reliability of the 2-BIT method for determining the effective order of nonlinear absorption, which confirms its efficacy.

Regarding the photoinitiator PIG, a different behavior is observed, with the best-fit exponent for *n* being 1.5. This is likely due to a combination of one- and two-photon absorption processes, as the PIG compound possesses electronic states that can be bridged by one or more photons under irradiation at 780 nm, consistent with the morphology of its absorption spectrum (Figure 4C,D). A possible interpretation of this case can be drawn from the works of Liaros et al., who suggested that single-photon absorption may lead to direct electronic transitions between lower singlet states of the molecule, which does not produce radicals, while two-photon absorption likely excites the molecule to a higher singlet excited state, where radical formation can occur.^47,48^ Additionally, excited state absorption (ESA) may contribute to the polymerization process when PIG is used. For ESA to occur, the excited-state lifetime must be longer than the pulse duration, allowing multiple photons to be absorbed before relaxation occurs. Since the excited-state lifetime of molecular systems can range from a few femtoseconds to microseconds, ESA could also provide a possible explanation of our result.^47^

In general, 2-BIT measurements demonstrated that all PIs, when integrated into the photoresist SZ2080, likely exhibit two-photon absorption behavior, making them a promising platform for efficient 3D structuring via MPL. These findings are further supported by Z-scan measurements and enriched where the PIs are analyzed as single molecules dissolved in solvents.

#### Z-Scan Measurements

Z-scan measurements were conducted on two different solution concentrations (0.5 and 1 mg/mL) of the synthesized compounds to accurately determine their TPA coefficients (β, Im γ, and σ). These coefficients offer insights into their effectiveness for MPL, as well as precise conditions for the most efficient energy-per-volume deposition. In Figure 6A, some representative OA Z- scans of 1 mg/mL HF- and LF-PIs solutions at varying laser intensities are shown, measured employing the same laser source as for 2-BIT (150 fs, 780 nm, 80 MHz). The solid lines represent the best fits of the experimental data points (indicated by solid symbols) by using eq 3. For comparison, similar experiments were conducted on BIS and SBB compounds, regarded as benchmark PIs for MPL due to their high efficiency and low fluorescence, respectively.^20^ The corresponding OA Z-scans of BIS and SBB solutions, measured under identical experimental conditions (i.e., concentrations, pulse width, and pulse intensities), are presented in Figure S1. The solvent showed negligible NLO absorption up to the maximum laser intensity of 3.0 GW/cm^2^ used for the measurements of the solutions. Because of the high laser repetition rate used for the Z-scan experiments, a temperature gradient in the refractive index, proportional to the linear absorption coefficient α_0_, could form, contributing to the NLO response of the compounds. However, given that studied compounds do not exhibit linear absorption at the laser excitation wavelength (except for PIG, which presents a very weak absorption), thermal energy accumulation can be regarded insignificant or, at most, a minor contributor.^49^ Therefore, the ultrafast (instantaneous) materials’ NLO response is primarily contributed by the electronic interactions with the laser radiation.

**Figure 6:**
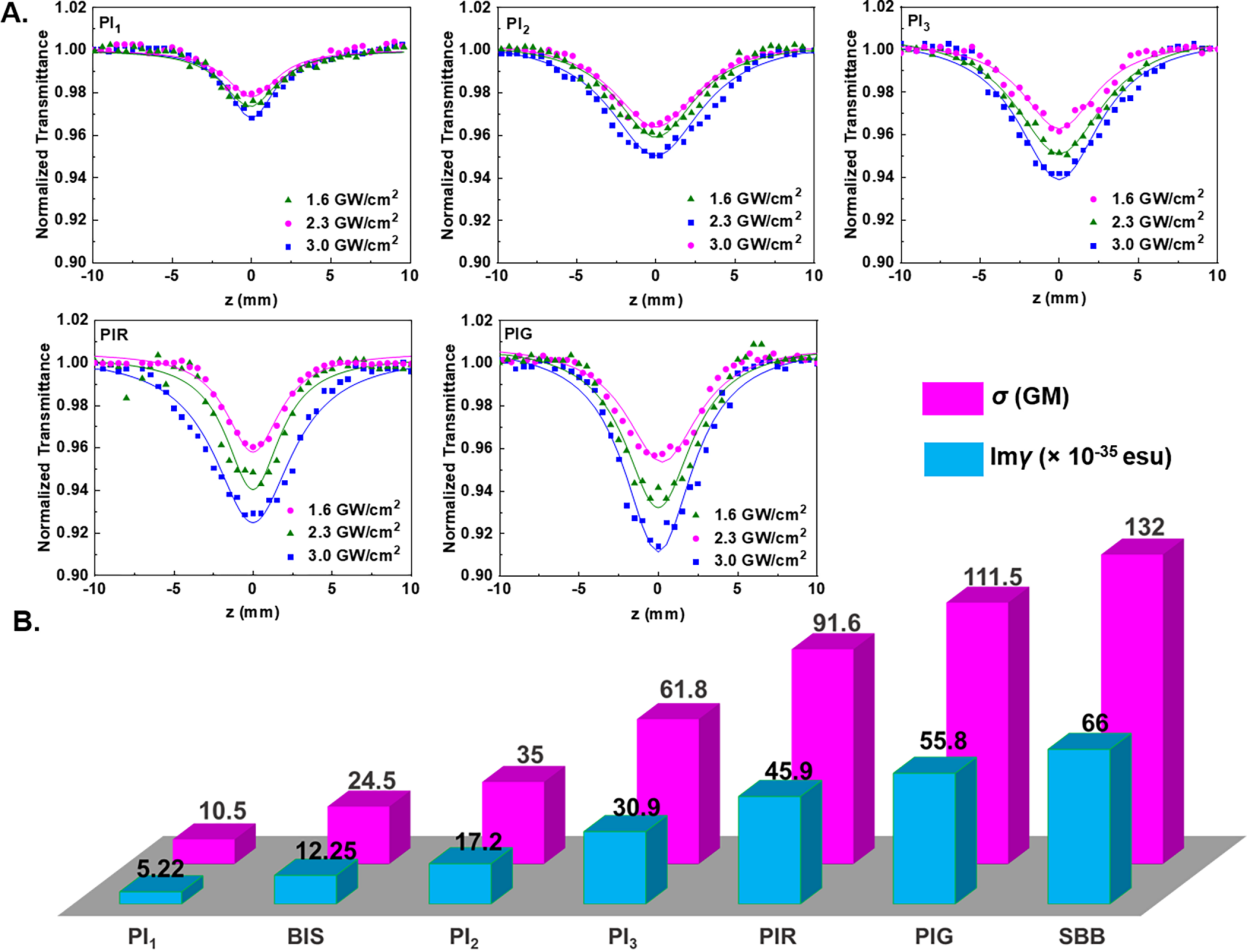
(A) OA Z-scans of HF- and LF-PIs: PI_1_ (6.6 mM), PI_2_ (4.6 mM), PI_3_ (6.1 mM), PIR (2.6 mM), and PIG (2.5 mM), under different laser excitation intensities. (B) Values of imaginary part of second-order hyperpolarizability (Im γ) and two-photon absorption cross section (σ) of HF- and LF-PIs, under 150 fs, 780 nm laser excitation. BIS and SBB are mentioned as references for HF-PIs and LF- PIs, respectively.

The Z-scan curves clearly show that all samples exhibit a transmission minimum near the focal plane, which increases with the pulse laser intensity. This shape of the OA Z-scan denotes the nonlinear absorption effect, i.e., a higher intensity pulse experiences higher absorption by the material, and corresponds to a reverse saturable absorption (RSA) behavior (where β > 0). Given the absence of any absorption features at the excitation wavelength of 780 nm, two- or multiphoton absorption could be considered as possible mechanisms to explain the pattern of the OA Z- scans. This mechanism is further supported by the existence of absorption bands in the UV spectral region (see absorption spectra of Figure 4B,C), which indicate that the electronic transitions require photon energies at least twice that of the photons at 780 nm to occur. However, as shown by the 2-BIT plots, and discussed in depth above, higher-order optical nonlinearities have a rather low probability of occurring in the studied compounds, with TPA being the primary process underlying their NLO response.^50^

From fitting the obtained OA Z-scan recordings with eq 3, the TPA absorption coefficients (β) of the compounds were determined. Then, the imaginary parts of their third-order susceptibility (Im χ^(3)^) and second-order hyperpolarizability (Im γ) along with their two-photon absorption cross sections (σ) were calculated by using eqs 4–6. The TPA coefficients determined for the different solution concentrations studied are gathered in Table S1. As shown in this table, the values of Im γ and σ remain unaffected by the molecular density, hence offering a figure of merit (FOM) for comparing the TPA values of the PIs. To facilitate comparisons, the FOM values for each compound are also presented in Figure 6B. From a simple inspection of this figure, two important conclusions can be drawn: (i) both LF-PIs and HF-PIs, except PI_1_, demonstrate significantly stronger NLO absorption than the high-fluorescent PI, BIS; (ii) the LF-PIs reveal comparable FOM values to those of the SBB. These findings underscore that HF-PIs based on triphenylamine-based and LF-PIs based on indane-1,3-dione could be as effective as BIS and SBB for initiating laser-induced polymerization.

By examination of the chemical structure of the compounds, valuable insights into the mechanisms responsible for their enhanced TPA properties can be gained. Among the HF-PIs, PI_3_ demonstrates enhanced NLO properties, ascribed to an improved electron delocalization resulting from the larger number of aromatic rings in its structure. This leads to a more effective charge distribution and increased molecular polarizability and thereby stronger NLO response. The stronger NLO response of PI_2_ compared to PI_1_ may be a result of Br substituent atoms. More precisely, bromine atoms behave as π-electron donor substituents, increasing the electron density within the π-electrons ring through resonance effects.^51^ This effect results in improved NLO response, as confirmed by DFT calculations reported elsewhere.^52^ Concerning the LF-PIs, which belong to the category of push–pull compounds, indane-1,3-dione acts as the electron-accepting group, while the electron-donating group varies, being either 2-thioxodihydro­pyrimidine in PIG or 2-butoxy-4-dimethylamino­benzene in PIR.^53–55^ Therefore, the larger TPA coefficients of PIG compared to those of PIR can be explained in terms of a more efficient charge transfer facilitated by the extended polyaromaticity of its electron-accepting group. This enhanced electron delocalization is confirmed by the more red-shifted absorption spectrum of the PIG.

To summarize, two methods were employed to determine the NLO response of the PIs. First, 2-BIT measurements, of the photoresist containing the PIs, indicated that the effective order of nonlinear absorption for all PIs was *n* ∼ 2 for all compounds except PIG, which exhibited intermediate behavior between one- and two-photon absorption. These results were further supported by Z- scan measurements, which confirmed that all PIs undergo excitation through TPA upon intense light excitation. Additionally, the Z-scan measurements showed that LF-PIs exhibited cross sections (σ) higher than those of HF- PIs. This may indicate an inverse correlation between fluorescence and nonlinear absorption efficiencies in these compounds, probably due to differing excited-state deactivation pathways, though no direct mechanistic relationship is implied. As high values of TPA cross-sections for PIs have been shown to correlate with greater efficiency in MPL,^9,56^ LF-PIs could enhance efficiency and sensitivity in MPL related technologies, due to their combined strong NLO response and low fluorescence. These results are demonstrated in the following sections, where cubes of LF-PIs are fabricated via MPL, and their fluorescence signals are investigated in comparison to those of HF-PIs and SBB.

### Photoluminescence Measurements of 3D Printed Microstructures

In Figure 7, the PL measurements of cubes fabricated via MPL (inset SEM images) are presented. These measurements confirm that the PL signal of HF-PIs (Figure 7A) significantly exceeds that of LF-PIs (Figure 7B). It is important to emphasize that the photoresist itself does not influence the PL signal, as it exhibits minimal emission in the visible spectral region.^29^

**Figure 7:**
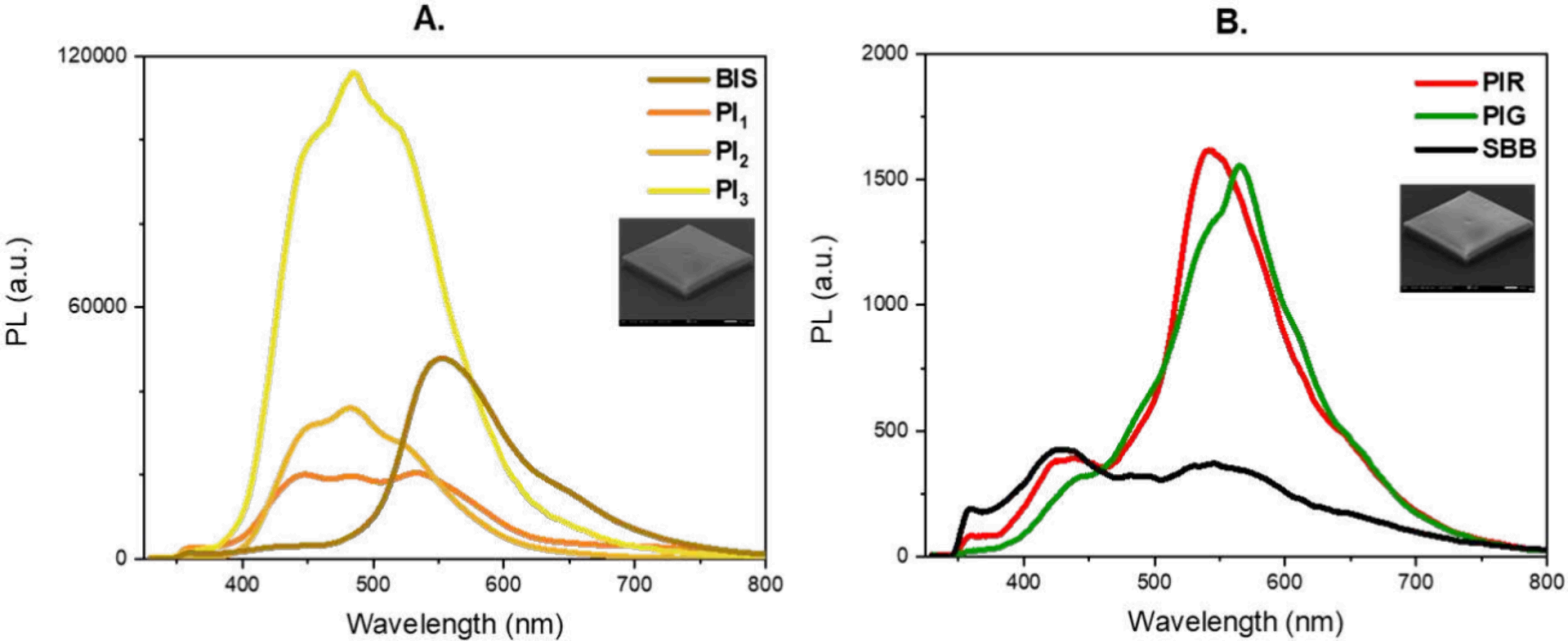
Photoluminescence measurements of the cubes (inserted SEM images) fabricated with (A) HF-PIs and (B) LF-PIs incorporated into SZ2080, excited with *P* = 35 mW at wavelength of 325 nm CW irradiation.

Regarding the HF- PIs, their PL intensities were compared with BIS. PI_1_ and PI_2_ exhibit similar PL intensities to BIS but demonstrate a PL signal 3 times lower than that of PI_3_. Notably, the HF-PIs display their main emission peak at approximately 485 nm, with PI_1_ featuring a broader emission spectrum ranging from 445 nm to 535 nm. Additionally, the HF- PIs PL peak is shifted to visible wavelengths, compared to their absorption spectra (Figure 4B), where it is clearly shown that in this wavelength the absorption of the HF-PIs is negligible. This can be attributed to energy losses (i.e., heat dissipation) during vibrational relaxation of the PIs’ excited states, a phenomenon known as Stokes shift,^57,58^ commonly observed in PIs.

Focusing on the LF-PIs, as they exhibit significantly stronger TPA than HF-PIs, their PL signals were comparable to SBB. Specifically, the LF-PIs exhibit a 2 orders of magnitude lower PL intensity than that of the HF-PIs, and a comparable PL signal to SBB. Furthermore, the LF-PIs display distinct PL peaks at 547 nm and 566 nm for PIR and PIG, respectively, in contrast to the broad PL spectrum of SBB, which ranges from 510 nm to 567 nm. It is worth mentioning that the PL peak for the substrate occurs at 437 nm (see Figure S2), which may explain the modest PL peak of SBB at 430 nm. A similar outcome was observed for the LF-PIs as for the HF-PIs, with both exhibiting PL peaks that are red-shifted relative to their absorption spectra (Figure 4 C), demonstrating the Stokes shift phenomenon.

Here, it is important to highlight that SBB is used exclusively in this work as a reference, as it has been widely studied as a low-fluorescence PI.^59^ However, recent multiphoton and harmonic imaging on 3D scaffolds containing SBB showed significant autofluorescence across different excitation wavelengths, which limits their suitability for bioapplications.^60^ This effect is primarily attributed to the broad absorption peak of SBB in the visible wavelength range, centered at around 600 nm (Figure 4C). In contrast, the new LF-PIs (i.e., PIR and PIG) do not show such pronounced characteristics (especially PIG), making them potentially better candidates for developing 3D microstructures intended for biorelated research that rely on excitation at specific wavelengths, particularly in nonlinear imaging approaches.^59^

### Fabrication of 3D Microstructures via MPL

The use of HF-PIs allows for the fabrication of well-defined 3D microstructures, as previously reported in ref 41. As such, they may be useful in applications that require high fluorescence signals, such as fluorescence microscopy, bioimaging, and related techniques.^60–62^

To demonstrate the suitability of LF-PIs for MPL, two distinct 3D microstructures were fabricated. In Figure 8A, the letters N, L, L (representing the abbreviation Non-Linear Lithography group at IESL-FORTH) were fabricated using the SZ2080^TM^ resist mixed with the LF-PIs and SBB. Figure 8B presents a 3D microstructure commonly employed as a scaffold for bioapplications,^63^ fabricated using the three LF-PIs, which are shown to be identical in geometry and well-resolved, proving their suitability as PIs for MPL and especially as scaffolds for bioapplications. Moreover, the 3D microstructures were printed at a scanning speed of 20 mm/s, with an average peak intensity of 0.42 TW/cm^2^ for PIG and PIR and 0.24 TW/cm^2^ for SBB.

**Figure 8:**
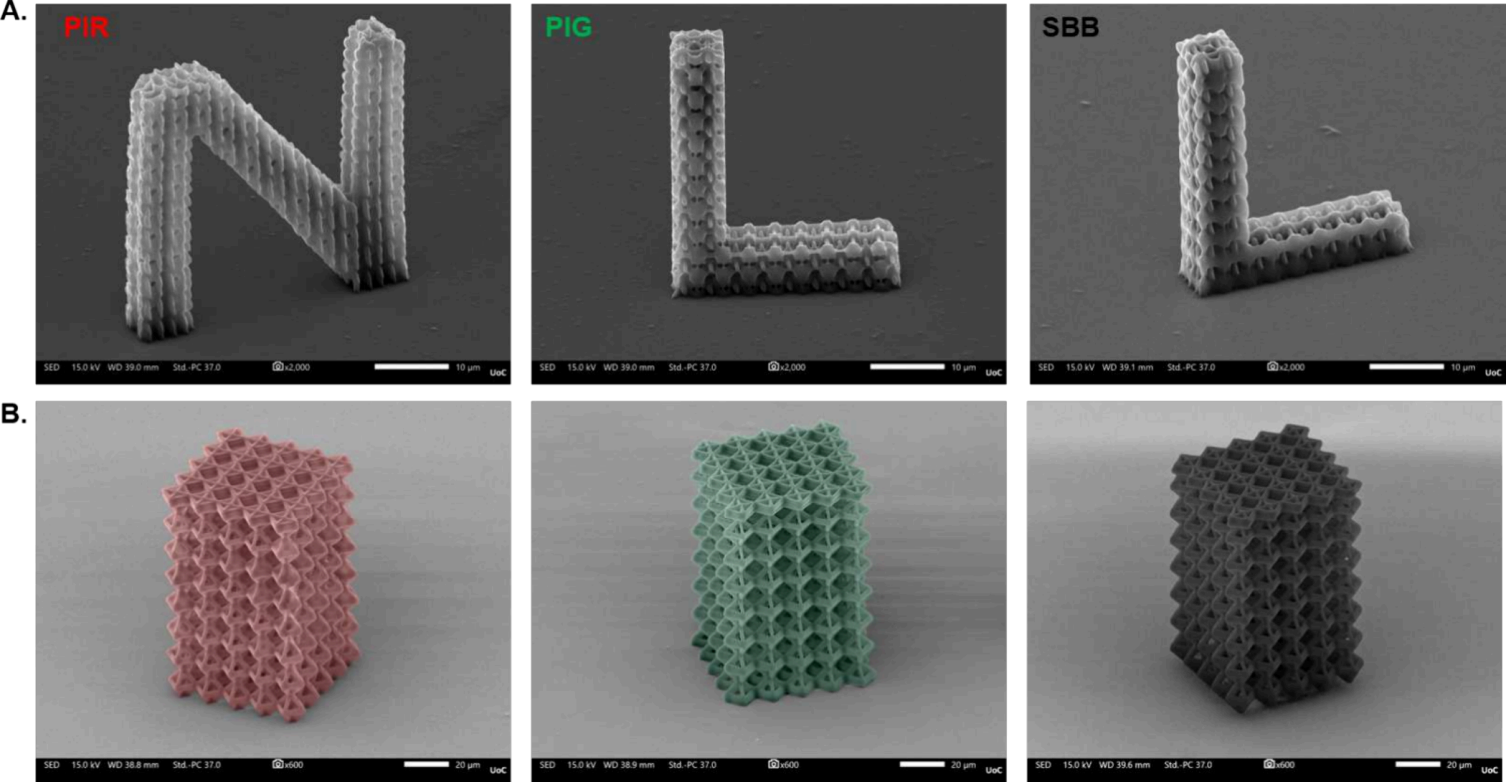
SEM images of 3D microstructures of LF-PIs. (A) 3D scaffolds shaped in the N (PIR), L (PIG), and L (SBB) letters. (B) 3D scaffolds of the same PIs in cubic shape. Red, green, and black are representing the intrinsic coloration of each PI.

Additionally, the scanning velocity used for fabricating the 3D microstructures of LF-PIs is similar to that typically used for standard PIs,^13^ making them a promising set of compounds for the MPL landscape, where low fluorescence is a drawback that needs to be mitigated. The fabrication of two different geometries using LF-PIs, combined with insights from their linear and nonlinear optical properties within a single optical system, demonstrates the versatility of these compounds. Moreover, it highlights the importance of determining the optical properties of PIs prior to the MPL process, as absorption, fluorescence signals, and NLO properties are strongly correlated to the effectiveness of MPL and the suitability of the results for specific applications.

## Conclusions

This study presents a unique approach that combines two well-established techniques for characterizing the NLO properties of PIs within a single optical system and for obtaining a qualitative picture of two-photon-induced energy deposition during MPL. To demonstrate the significance of this approach, the effectiveness of triphenylamine-based aldehydes with varying aromaticity and indane-1,3-dione-based push–pull compounds as high- and low-fluorescent PIs for MPL was investigated, respectively. Specifically, the measured nonlinear coefficients of LF-PIs, reaching values up to 10 times greater than those of HF-PIs, highlight their superior suitability for MPL. This enhanced effectiveness was attributed to their ability to minimize radiative relaxation through efficient charge transfer processes, leading to strong TPA nonlinearities. Notably, the LF-PI PIG, introduced for the first time, was showcased as an alternative for SBB in MPL, because it enabled the fabrication of 3D microstructures with narrow fluorescence signal.

Our findings may pave the way for future research focused on efficient MPL 3D microstructuring with minimal fluorescence, which is of high importance for applications in advanced optics, sensitive bioapplications, and beyond. The demonstrated approach of integrating material NLO characterization and MPL-based fabrication under similar spectrotemporal regimes provides a practical and efficient methodology for accelerating advancements in MPL related technologies. Future work could focus on combining this approach with a tunable femtosecond oscillator to investigate the NLO properties of PIs at different wavelengths. This could potentially lead to the development of new PIs optimized for broader spectral ranges and provide deeper insights into the fundamental physics governing the MPL process.

## Supplementary Material



## Notes

Z-scan recordings of BIS and SBB compounds; NLO absorption-related parameters of different concentrations; photoluminescence measurements of the glass substrate; synthesis of the PIs (PDF)
