# Supplementary material for: High- and Low-Fluorescent Photoinitiators for Multiphoton Lithography
Source: ACS Appl Polym Mater. 2025 Jul 31;7(15):10108–20. doi: 10.1021/acsapm.5c01802 (PMC12340759; doi:10.1021/acsapm.5c01802)
Supplement: Supplementary file 1 [file ap5c01802_si_001.pdf]

Supporting Information

High- and Low-Fluorescent Photoinitiators for Multi-Photon Lithography

Dimitra Ladika,[1,2] Michalis Stavrou,[1] Gordon Zyla,[1,2] Kostas Parkatzidis,[3] Maria Androulidaki,[1] Frederic Dumur,[4] Maria Farsari,[1] and David Gray[1*]*

[1]Institute of Electronic Structure and Laser, Foundation for Research and Technology-Hellas, 70013 Heraklion, Greece

[2]Laser Research Center, Physics Faculty, Vilnius University, Vilnius, LT-10223 Lithuania

[3]Department of Chemical Engineering, Stanford University, Stanford, CA 94305, USA

[4]UMR 7273, Aix Marseille University, CNRS, ICR, 13397 Marseille, France

Corresponding authors:

* Dimitra Ladika (email : dladika@iesl.forth.gr)

* David Gray (email: dgray@iesl.forth.gr)

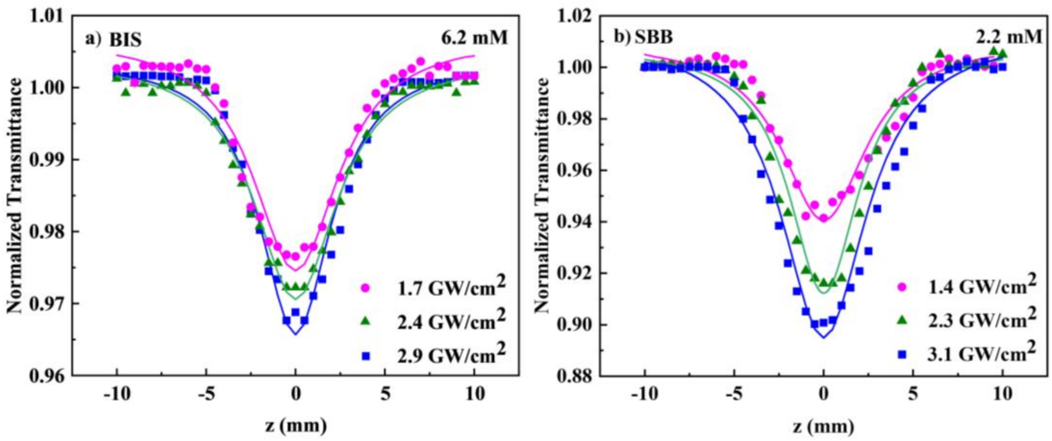

Figure S1. OA Z-scans of (a) BIS (6.6 mM) and (b) SBB compounds, under different laser excitation intensities.

Table S1. Nonlinear absorption-related parameters (β: nonlinear absorption coefficient, Im$\chi^{(3)}$: imaginary part of the third-order susceptibility, Imγ: imaginary part of second-order hyperpolarizability, σ: two-photon absorption cross section) of different concentration solutions of BIS, SBB, PI $_{(1,2,3)}$, PIR and PIG determined by Z-scan, under 150 fs, 780 nm laser excitation.

Samples	C (mM)	β (x 10^{-11} cm/W)	Im$\chi^{(3)}$ (x 10^{-14} esu)	Imγ (x 10^{-35} esu)	σ (GM)
BIS	3.1	1.8 ± 0.6	0.9 ± 0.1	12.1 ± 0.4	24.3 ± 1.0
	6.2	3.8 ± 0.5	1.9 ± 0.2	12.4 ± 2.0	24.8 ± 3.0
SBB	1.1	3.5 ± 0.1	1.8 ± 0.1	64.3 ± 3.0	129 ± 5
	2.2	7.4 ± 0.3	3.7 ± 0.2	67.6 ± 3.0	135 ± 6
PI_1	6.6	1.8 ± 0.2	0.9 ± 0.1	5.4 ± 0.5	10.7 ± 1.0
	13.2	3.2 ± 0.5	1.6 ± 0.2	5.0 ± 0.1	10 ± 1
PI_2	4.6	4.2 ± 0.2	2.1 ± 0.1	18.3 ± 1.0	37 ± 2
	9.2	7.4 ± 0.3	3.7 ± 0.1	16.2 ± 0.6	33 ± 1
PI_3	3.05	4.7 ± 0.2	2.4 ± 0.8	31.01 ± 1.0	62 ± 2
	6.1	9.3 ± 0.6	4.7 ± 0.3	30.8 ± 2.0	61.6 ± 4.0
PIR	2.6	5.7 ± 0.4	2.9 ± 0.2	44.4 ± 3.0	88.8 ± 6.0
	5.3	12.4 ± 0.5	6.3 ± 0.2	47.3 ± 2.0	94.5 ± 7.0
PIG	1.7	4.5 ± 0.5	2.3 ± 0.3	54.9 ± 6.0	110 ± 10
	2.5	7.0 ± 0.7	3.5 ± 0.3	56.7 ± 5.0	113 ± 10

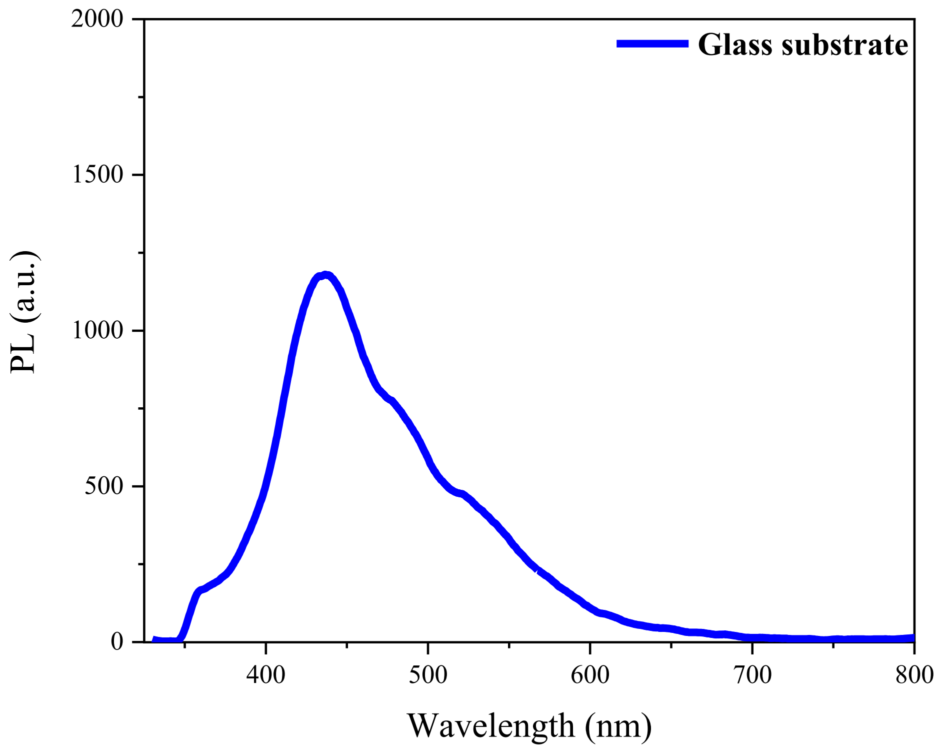

Figure S2. Photoluminescence measurements of the glass substrate used for the cubes fabrication, excited with P=35mW at wavelength of 325 nm CW irradiation.

Synthesis of the PIs

Synthesis of 4,4'-(phenylazanediyl)dibenzaldehyde [PI_1]

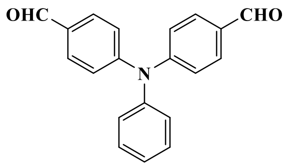

Chemical Formula: $C_{20}H_{15}NO_2$
Molecular Weight: 301.3450

$POCl_3$ (36 mL) was added dropwise to DMF (70 mL) with stirring at 0°C and the stirring was maintained at this temperature for 15 min. Then triphenylamine (20 g, 81.53 mmol, M = 245.32 g/mol) was added in one portion and the mixture was stirred at 135°C overnight. The reaction mixture was poured into ice, the yellow solid was then filtered, dissolved in DCM, dried over magnesium sulfate and the solvent removed under reduced pressure. The residue was chromatographied over silicagel using DCM / acetone (4/1) as the eluent. A bright yellow solid was obtained in 85% yield (20.88 g).

^1H NMR (300 MHz, $CDCl_3$) δ 9.90 (s, 2H), 7.85 – 7.72 (m, 4H), 7.40 (dd, J = 8.4, 7.0 Hz, 2H), 7.24 – 7.13 (m, 7H).

^{13}C NMR (75 MHz, $CDCl_3$) δ 190.59, 152.19, 145.71, 131.53, 131.45, 130.31, 129.88, 127.23, 126.48, 126.42, 122.95.

HRMS (ESI MS) m/z: theory: 302.1176, found: 302.1179 ((M+H)+ detected)

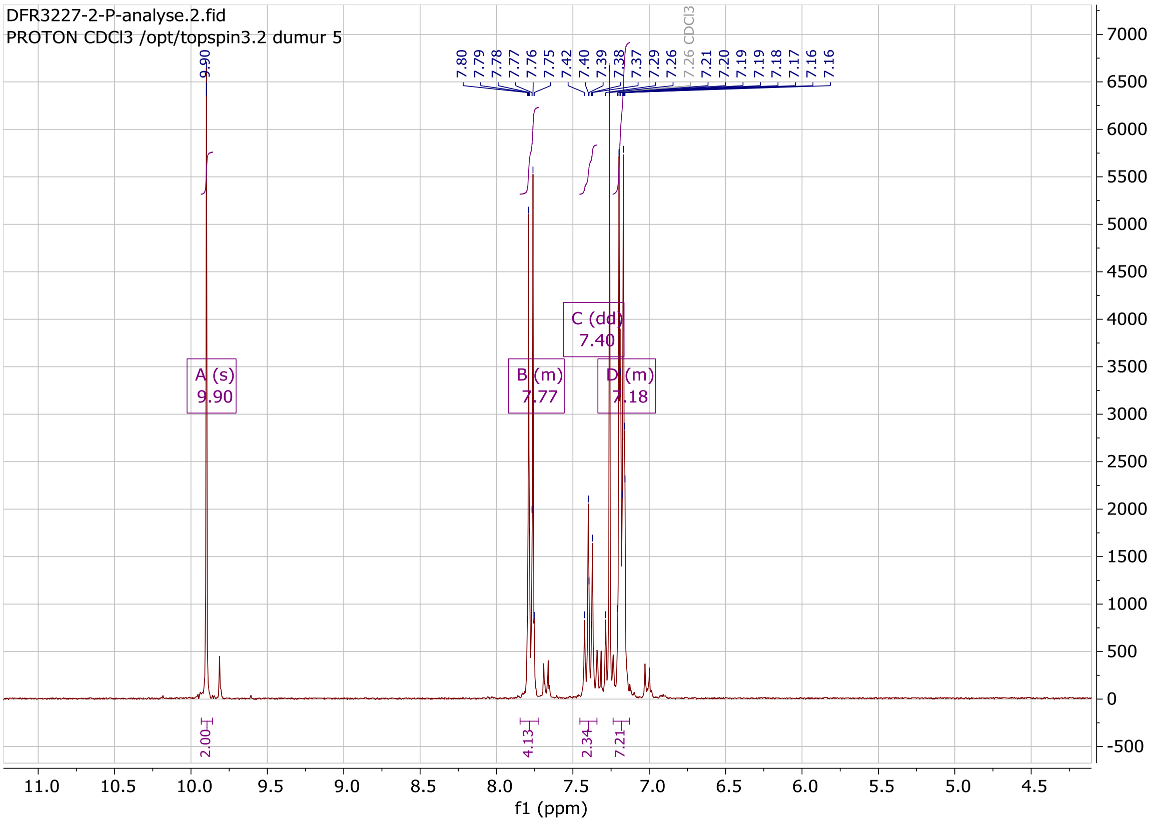

Figure S3. ^1H NMR spectrum of 4,4'-(phenylazanediyl)dibenzaldehyde [PI_1]

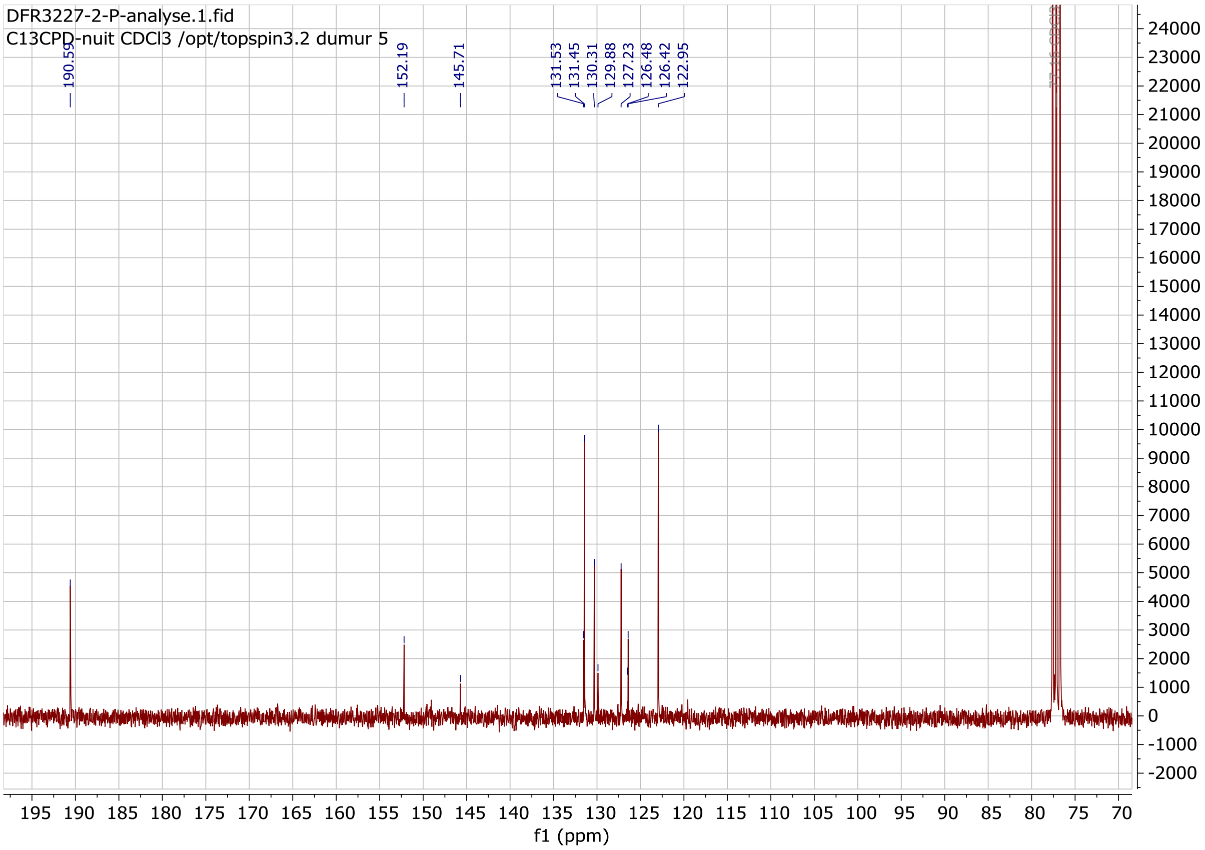

Figure S4. ^{13}C NMR spectrum of 4,4'-(phenylazanediyl)dibenzaldehyde [PI_1]

Synthesis of 4-(*bis*(4-bromophenyl) amino) benzaldehyde [PI_2]

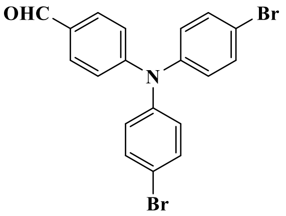

Chemical Formula: $C_{19}H_{13}Br_2NO$
Molecular Weight: 431.1270

To a solution of 4-(*N,N*-diphenylamino)benzaldehyde (27.3 g, 10 mmol, M = 273.33 g/mol) dissolved in DMF (250 mL) was slowly added *N*-bromosuccinimide (NBS) (35.59 g, 20 mmol, M = 177.98 g/mol) dissolved in DMF (150 mL). After stirring overnight, DMF was removed under reduced pressure. The residue was suspended in chloroform. The organic phase was washed several times with water, dried over magnesium sulfate and the solvent removed under reduced pressure. The product was purified by column chromatography (SiO_2) using a mixture of chloroform/pentane 3/1 as the eluent. The product was recrystallized in methanol and obtained as a light yellow solid (98% yield, 42.25 g).

^{1}H NMR (400 MHz, $CDCl_3$) δ 9.84 (s, 1H), 7.75 – 7.67 (m, 2H), 7.49 – 7.41 (m, 4H), 7.09 – 6.97 (m, 6H).

^{13}C NMR (101 MHz, $CDCl_3$) δ 190.52, 152.53, 145.19, 133.10, 131.53, 130.35, 127.55, 120.61, 118.25.

HRMS (ESI MS) m/z: theory: 429.9437 found: 429.9434 ((M+H)+ detected)

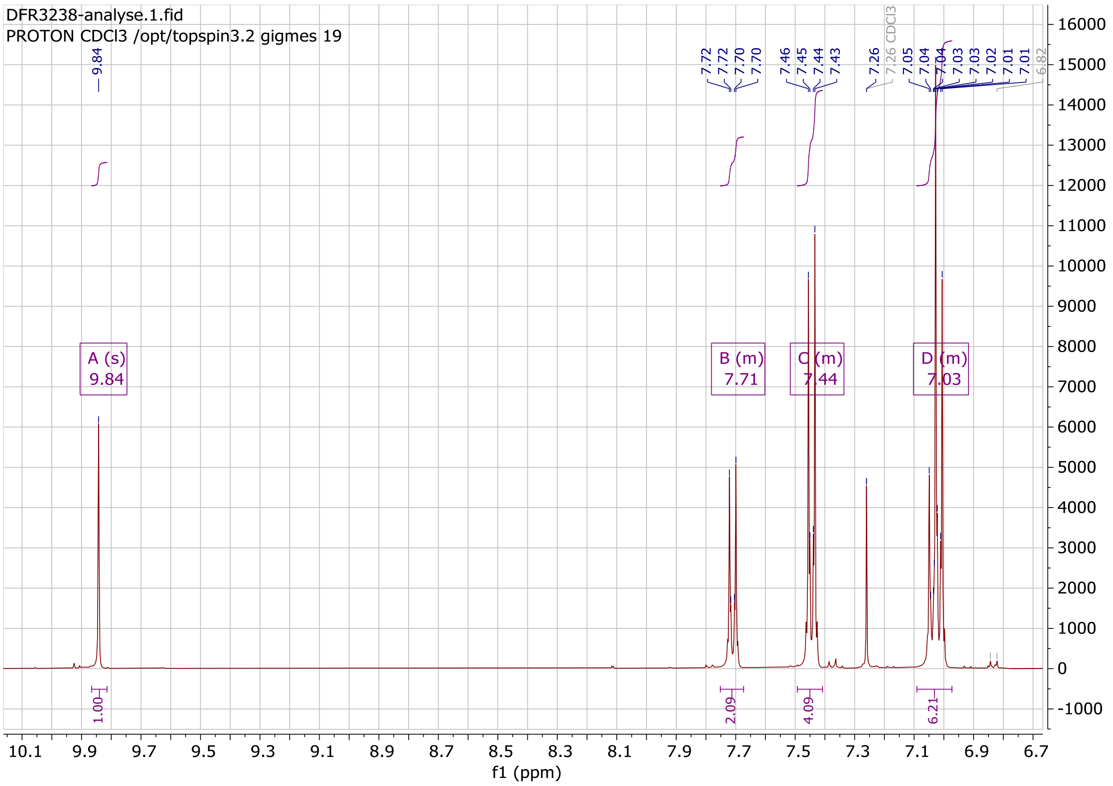

Figure S5. ^{1}H NMR spectrum of 4-(*bis*(4-bromophenyl) amino) benzaldehyde [PI_2]

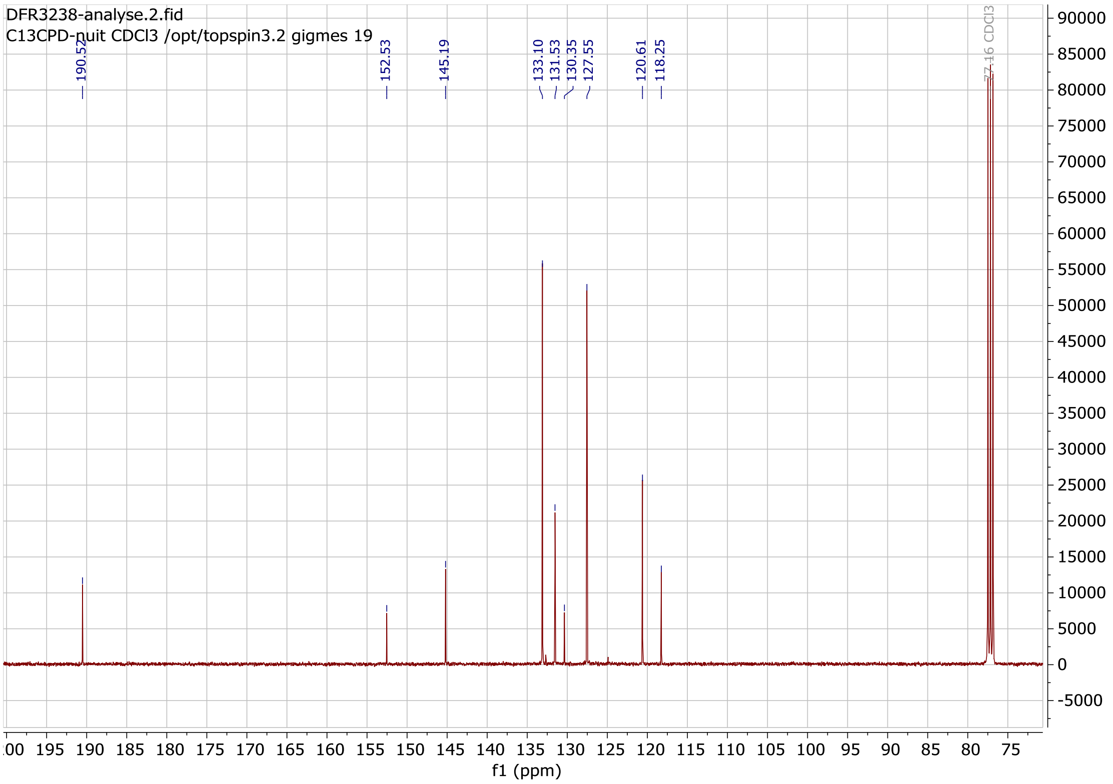

Figure S6. ^{13}C NMR spectrum of 4-(bis(4-bromophenyl) amino) benzaldehyde [PI_2]

Synthesis of 4',4'''-((4-formylphenyl)azanediyl)bis(([1,1'-biphenyl]-4-carbaldehyde)) [PI_3]

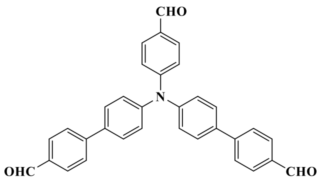

Chemical Formula: $C_{33}H_{23}NO_3$
Molecular Weight: 481.5510

Tetra*kis*(triphenylphosphine)palladium (0) (0.46 g, 0.744 mmol, M = 1155.56 g.mol^{-1}) was added to a mixture of 4-(*bis*(4-bromophenyl)amino)benzaldehyde (2.52 g, 6.11 mmol, M = 413.13 g.mol^{-1}), (4-formylphenyl)boronic acid (1.90 g, 12.66 mmol, M = 149.94 g.mol^{-1}), toluene (54 mL), ethanol (26 mL) and an aqueous potassium carbonate solution (2 M, 6.91 g in 25 mL water, 26 mL) under vigorous stirring. The mixture was stirred at 80 °C for 48 h under a nitrogen atmosphere. After cooling to room temperature, the reaction mixture was poured into water and extracted with ethyl acetate. The organic layer was washed with brine several times, and the solvent was then evaporated. Addition of DCM followed by pentane precipitated a white solid which was filtered off. The residue was purified by column chromatography (SiO_2, pentane/DCM: 1/1 and pure DCM) and isolated as a solid (88% yield, 2.59 g).

^{1}H NMR (300 MHz, $CDCl_3$) δ 10.06 (s, 2H), 9.88 (s, 1H), 8.02 – 7.91 (m, 4H), 7.82 – 7.72 (m, 6H), 7.70 – 7.58 (m, 4H), 7.34 – 7.25 (m, 4H), 7.20 (d, J = 8.7 Hz, 2H).

^{13}C NMR (75 MHz, $CDCl_3$) δ 191.84, 190.54, 152.74, 146.65, 146.19, 136.25, 135.41, 132.33, 132.20, 131.52, 130.59, 130.52, 128.84, 128.74, 128.58, 127.45, 126.27, 121.38.

HRMS (ESI MS) m/z: theory: 482.1751 found: 482.1758 ((M+H)+ detected)

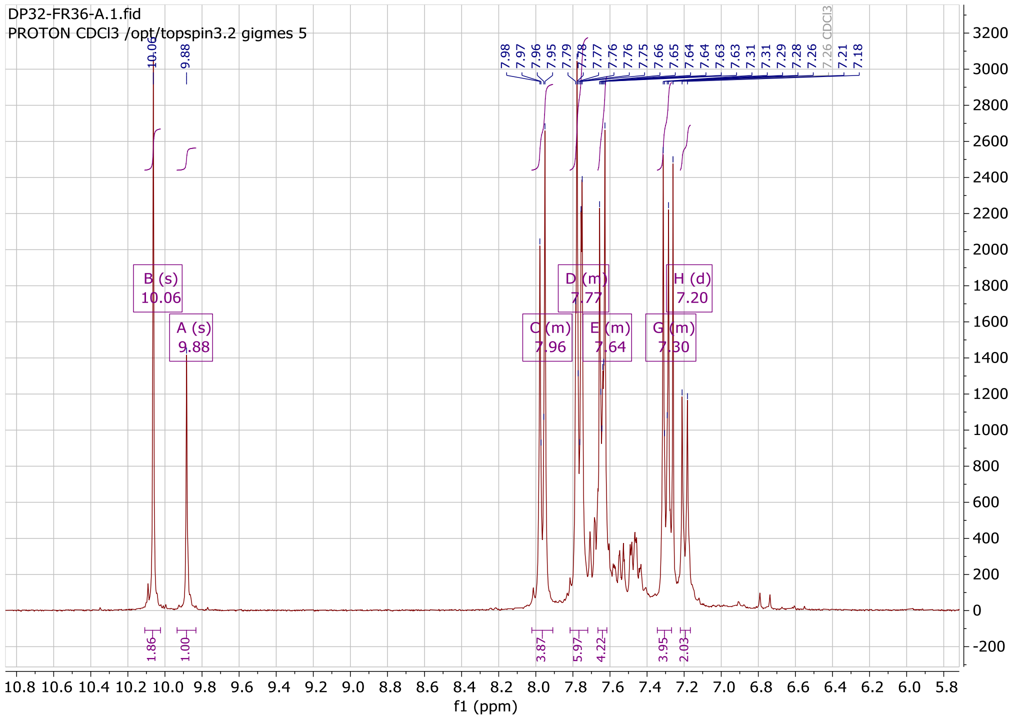

Figure S7. ^{1}H NMR spectrum of 4',4'''-((4-formylphenyl)azanediyl)bis(([1,1'-biphenyl]-4-carbaldehyde)) [PI_3]

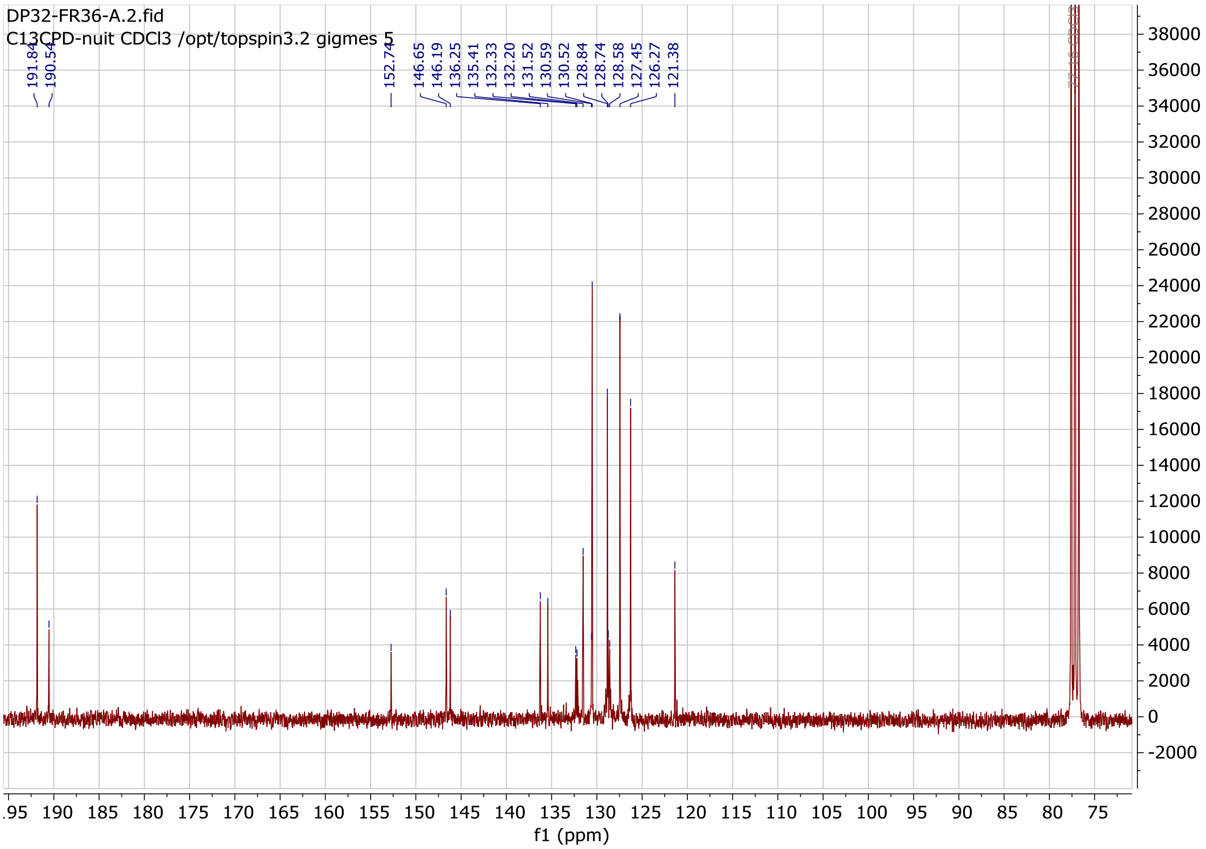

Figure S8. ^{13}C NMR spectrum of 4',4'''-((4-formylphenyl)azanediyl)bis(([1,1'-biphenyl]-4-carbaldehyde)) [PI3]

Synthesis of 2-(2-butoxy-4-(diethylamino) benzylidene)-1*H*-indene-1,3(2*H*)-dione [PIR]

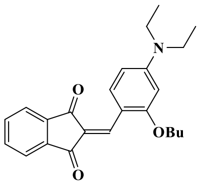

Chemical Formula: $C_{24}H_{27}NO_3$
Molecular Weight: 377.4840

Indane-1,3-dione (0.37 g, 2.55 mmol, M = 146.14 g/mol) and 2-butoxy-4-(diethylamino) benzaldehyde (0.64 g, 2.55 mmol, M = 249.35 g/mol) were dissolved in absolute ethanol (50 mL) and a few drops of piperidine were added. The reaction mixture was refluxed and progress of the reaction was followed by TLC. After cooling, a precipitate formed. It was filtered off, washed several times with ethanol and dried under vacuum (92% yield, 0.88 g).

^1H NMR (300 MHz, $CDCl_3$) δ 9.50 (d, J = 9.3 Hz, 1H), 8.58 (s, 1H), 8.34 (s, 2H), 8.04 (dd, J = 6.2, 3.3 Hz, 2H), 7.61 (dt, J = 6.3, 3.5 Hz, 2H), 6.43 (dd, J = 9.3, 2.4 Hz, 1H), 6.04 (d, J = 2.4 Hz, 1H), 4.08 (t, J = 6.4 Hz, 2H), 3.50 (q, J = 7.1 Hz, 4H), 2.03 – 1.83 (m, 2H), 1.74 – 1.53 (m, 3H), 1.28 (t, J = 7.1 Hz, 6H), 1.04 (t, J = 7.3 Hz, 3H).

^{13}C NMR (75 MHz, $CDCl_3$) δ 192.00, 189.93, 164.29, 155.37, 142.17, 138.19, 137.98, 136.44, 136.29, 136.25, 130.35, 130.26, 128.37, 128.28, 123.34, 122.55, 122.52, 113.35, 105.26, 93.29, 68.43, 45.29, 31.28, 19.57, 14.04, 12.98.

HRMS (ESI MS) m/z: theory: 378.2064 found: 378.2071 ((M+H)+ detected)

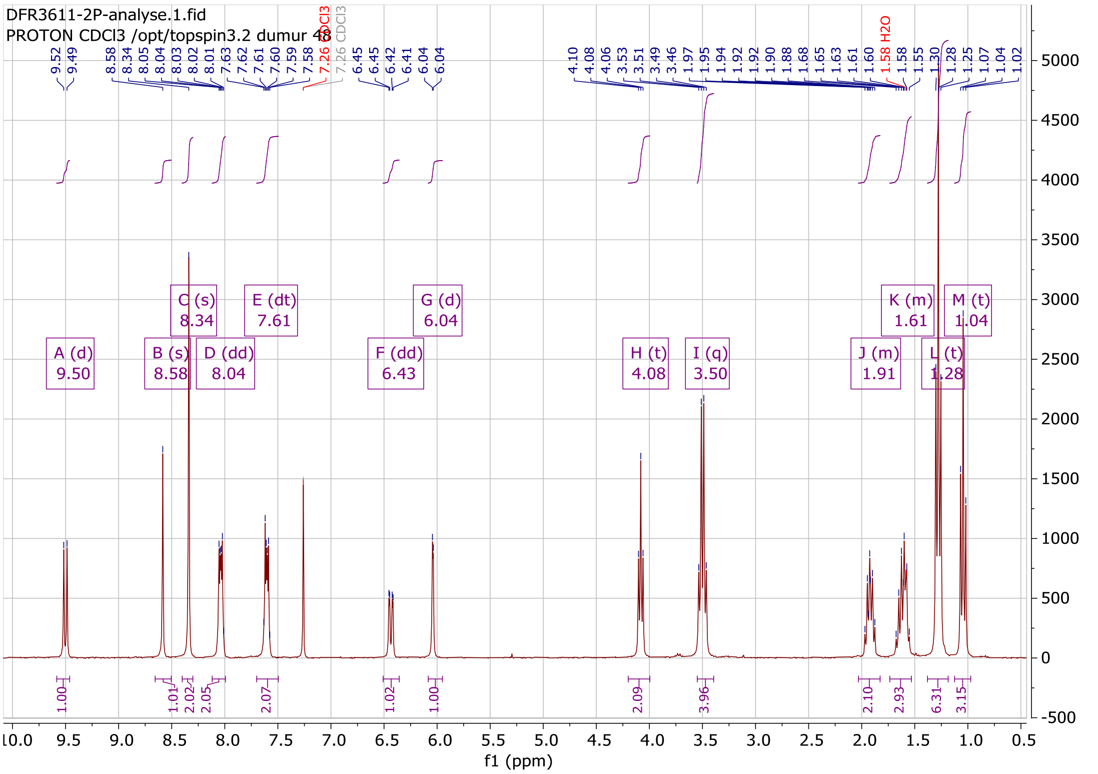

Figure S9. ^{1}H NMR spectrum of 2-(2-butoxy-4-(diethylamino) benzylidene)-1*H*-indene-1,3(2*H*)-dione [PIR]

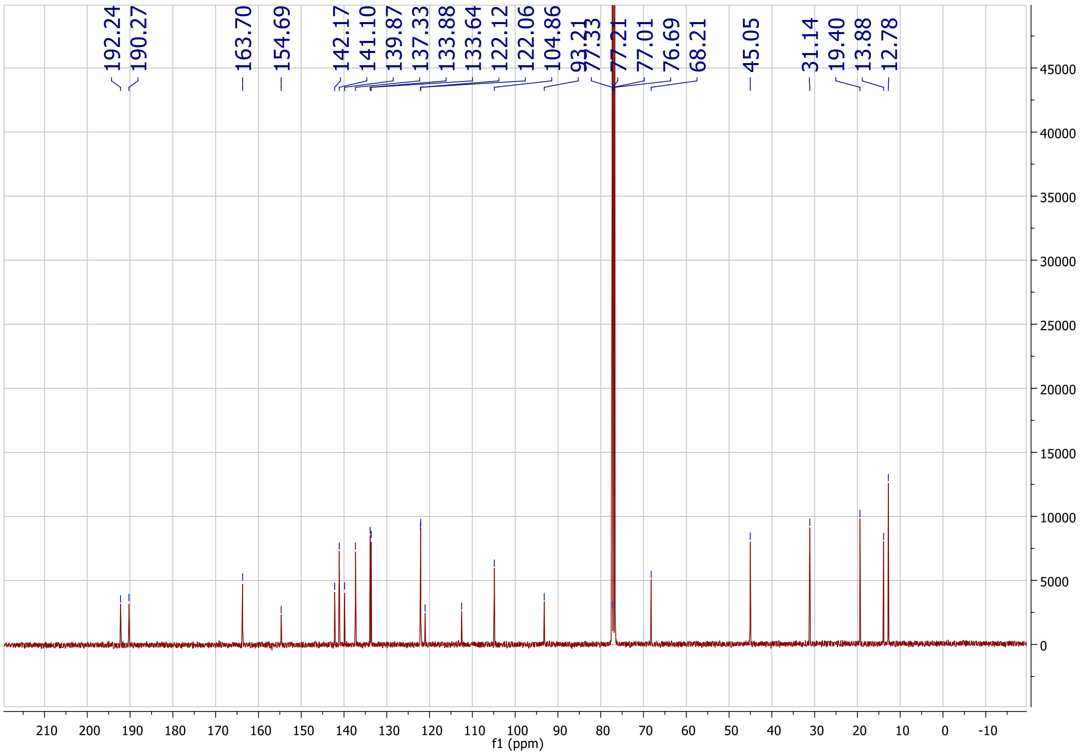

Figure S10. ^{13}C NMR spectrum of 2-(2-butoxy-4-(diethylamino) benzylidene)-1*H*-indene-1,3(2*H*)-dione [PIR]

Synthesis of 5-(1',3'-dioxo-1',3'-dihydro-[1,2'-biindenylidene]-3(2*H*)-ylidene)-1,3-diethyl-2-thioxodihydropyrimidine-4,6(1*H*,5*H*)-dione [PIG]

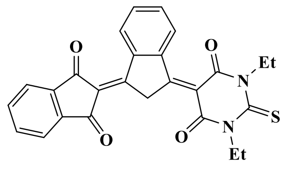

Chemical Formula: $C_{26}H_{20}N_2O_4S$
Molecular Weight: 456.5160

Indane-1,3-dione (2 g, 13,68 mmol, M = 146.15 g/mol) and 1,3-diethyl-2-thiobarbituric acid (2.74 g, 13,68 mmol, M = 200.26 g/mol) were suspended in absolute ethanol (50 mL) and a few drops of piperidine were added. The solution was introduced in a preheated bath at 100°C and the solution was refluxed overnight. During reflux, a yellow precipitate formed. The solution was cooled to room temperature. The solution was acidified with diluted aqueous HCl. The yellow solid was filtered off, washed several times with ethanol, then pentane and dried under vacuum (43% yield). NMR analyses were performed in DMSO-d_6 in which only the anionic form of PIG was detected, as classically observed for strong electron acceptors in this solvent.

^{1}H NMR (300 MHz, DMSO) δ 8.96 – 8.85 (m, 1H), 8.32 (s, 1H), 7.82 (s, 4H), 7.51 – 7.40 (m, 1H), 7.22 – 7.04 (m, 2H), 4.47 (q, J = 6.8 Hz, 4H), 1.20 (t, J = 6.8 Hz, 6H).

^{13}C NMR (75 MHz, DMSO) δ 190.89, 175.59, 159.68, 159.45, 158.40, 144.26, 140.87, 138.05, 134.52, 130.17, 127.50, 126.88, 125.42, 123.24, 121.73, 117.92, 92.74, 43.76, 41.92, 22.19, 21.57, 12.70.

Elemental analysis Calc: C, 68.4; H, 4.4; O, 14.0; Found: C, 68.7; H, 4.6; O, 13.8

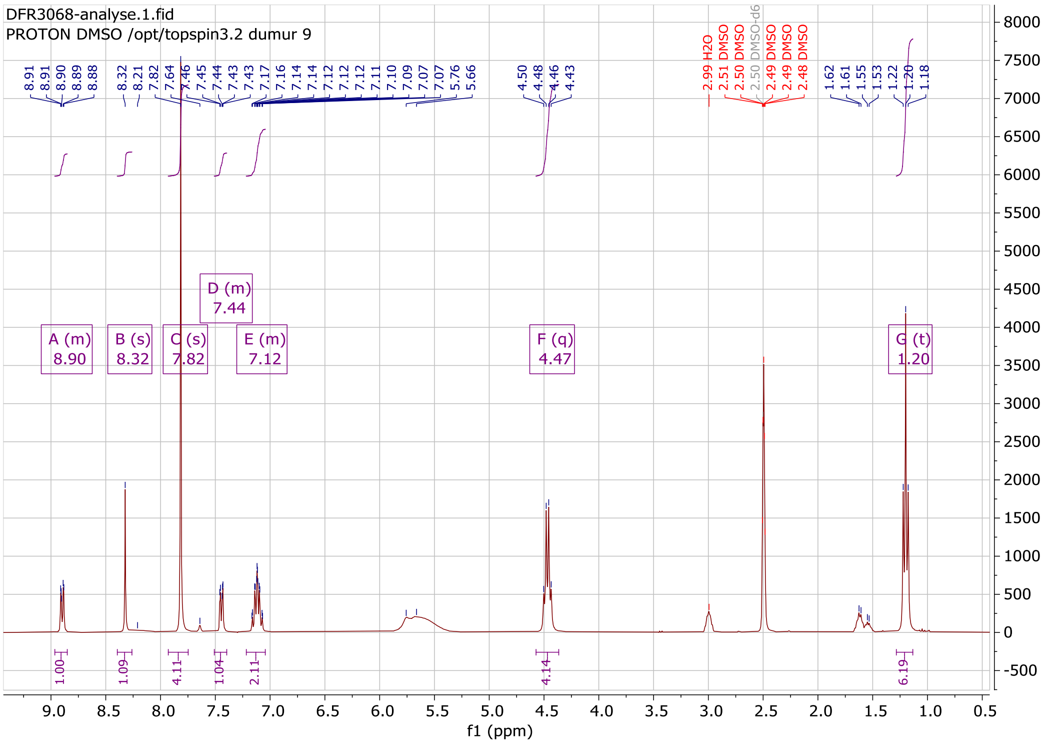

Figure S11. ^{1}H NMR spectrum of 5-(1',3'-dioxo-1',3'-dihydro-[1,2'-biindenylidene]-3(2*H*)-ylidene)-1,3-diethyl-2-thioxodihydropyrimidine-4,6(1*H*,5*H*)-dione [PIG]

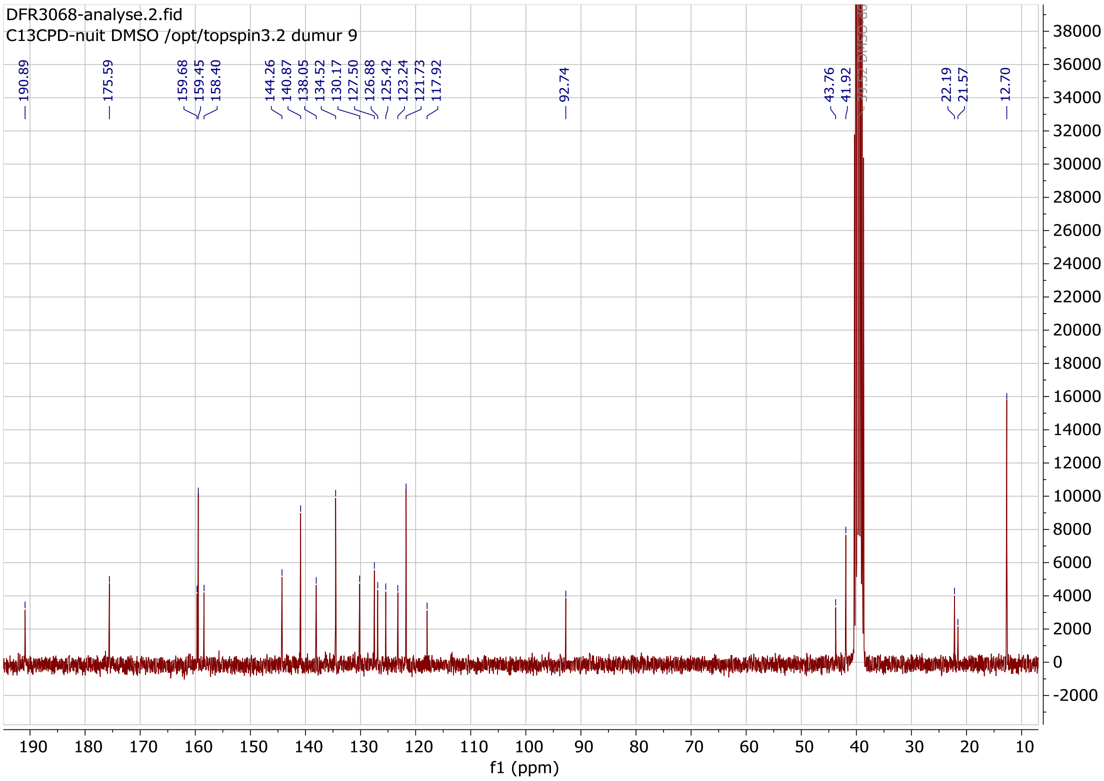

Figure S12. ^{13}C NMR spectrum of 5-(1',3'-dioxo-1',3'-dihydro-[1,2'-biindenylidene]-3(2*H*)-ylidene)-1,3-diethyl-2-thioxodihydropyrimidine-4,6(1*H*,5*H*)-dione [PIG]
